# Supplementary material for: Application of a new highly multiplexed amplicon sequencing tool to evaluate Plasmodium falciparum antimalarial resistance and relatedness in individual and pooled samples from Dschang, Cameroon
Source: Front Parasitol. 2025 Feb 5;3:1509261. doi: 10.3389/fpara.2024.1509261 (PMC11835963; doi:10.3389/fpara.2024.1509261)
Supplement: Supplementary file 2 [file DataSheet1.pdf]

Supplemental Material

Table of Contents

Figure S1 2
Figure S2 3
Figure S3 4
Figure S4 5
Figure S5 6
Figure S6 7
Figure S7 8
Figure S8 9
Figure S9 10
Figure S10 12
Figure S11 13
Figure S12 14
Figure S13 ....,,15
Table S1 16
Table S2 18
Table S3 19
Table S4 20
Table S5 22
Table S6 24
Table S7 25
Table S8 26
Table S9 28
Table S10 29
Table S11 30
Table S12 31
Table S13 32
Supplementary Data Sheet 1. Protocol 1: *Pf*-SMARRT Amplification 35
Supplementary Data Sheet 2. Protocol 2: Library preparation 39
Data Availability 43

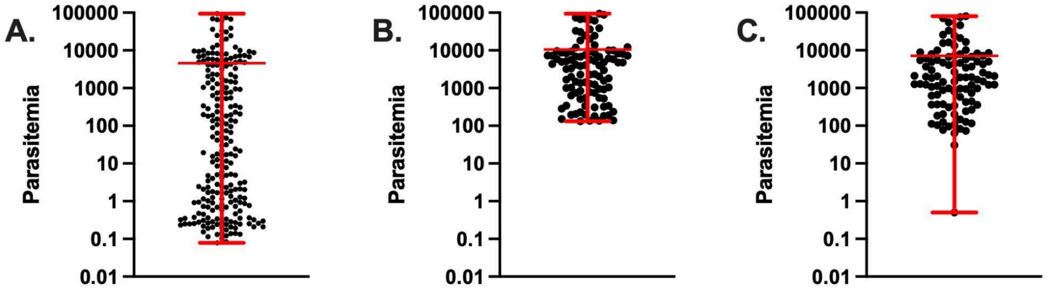

Figure S1. PCR estimated parasitemias based on real-time PCR. Panel A shows the distribution of estimated parasitemia in the entire study population. Panel B shows the estimated parasitemias of the 100 selected for this sequencing study. Panel C shows the estimated parasitemia after re-extraction. Red bars show mean and whiskers denote maximum and minimum parasitemia. Parasitemia is shown on a log10 scale.

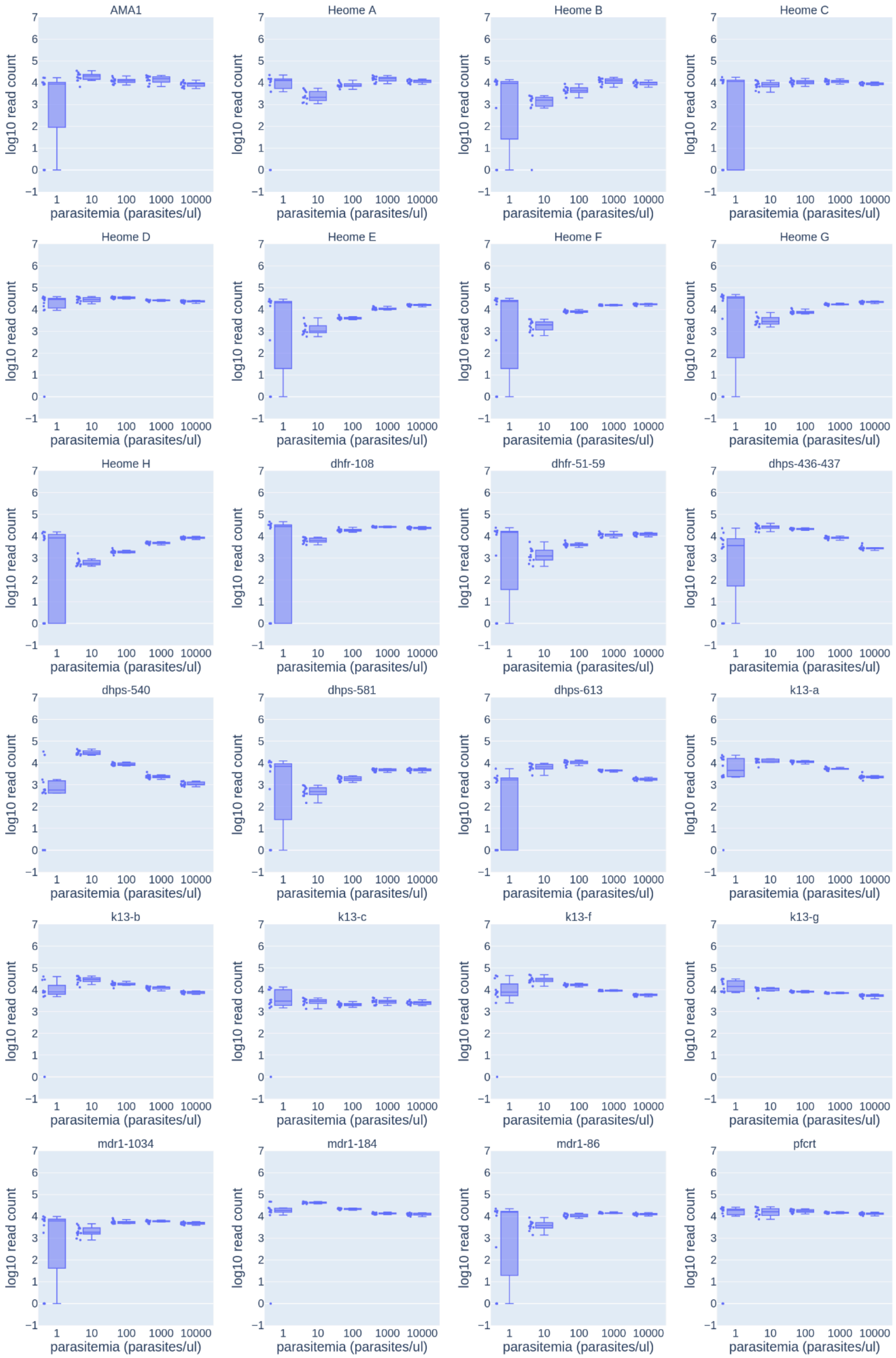

Figure S2. Sequencing depth for assay development controls. Plots depict sequencing depth on a log10 scale for 12 samples at each parasitemia. At each parasitemia level, individual points for values are plotted (left) and a box and whiskers plot with maximum and minimum values (right).

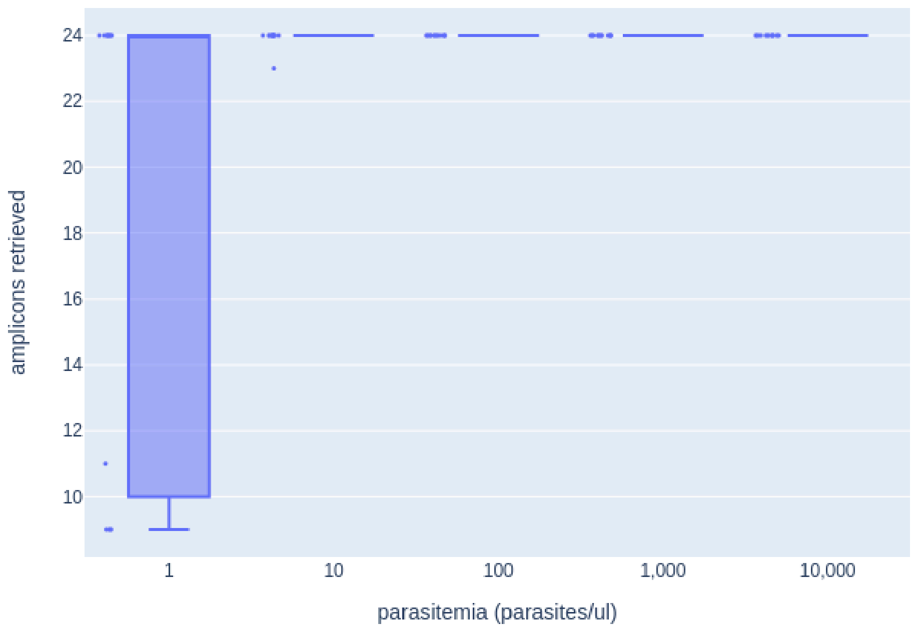

Figure S3. Number of successfully genotyped amplicons in controls at different parasitemias. Successfully genotyped amplicons are defined as amplicons that retrieve at least one haplotype within a given replicate. At each parasitemia level, individual points for values are plotted (left) and a box and whiskers plot with maximum and minimum values (right).

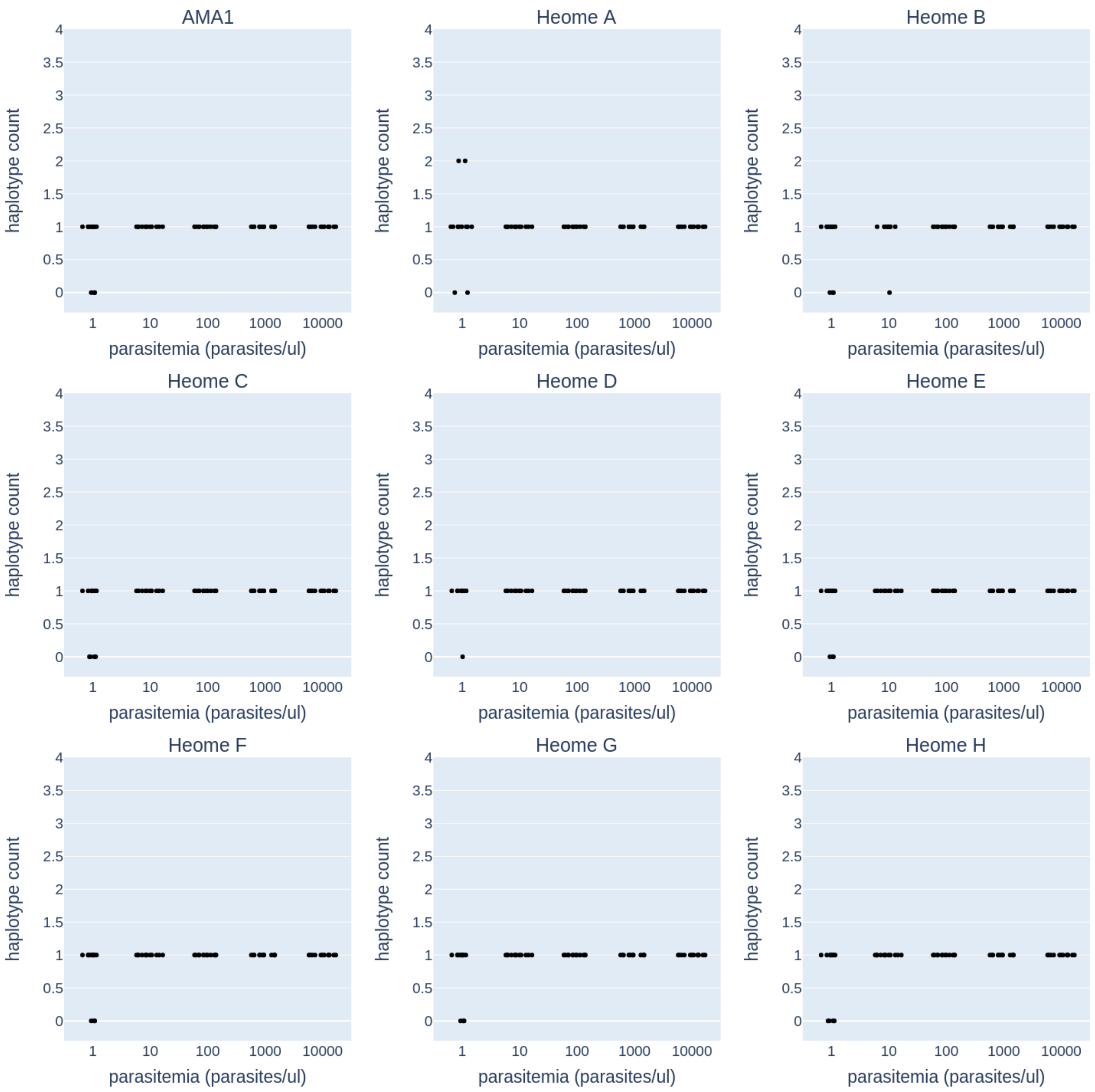

Figure S4. Haplotype counts in monoclonal control samples at different parasitemias for diversity amplicons. This figure shows the relationship between parasitemia and the number of haplotypes detected for amplicons that are markers of diversity. There are 12 samples at each parasitemia, and because the samples are monoclonal, the expected number of haplotypes is one.

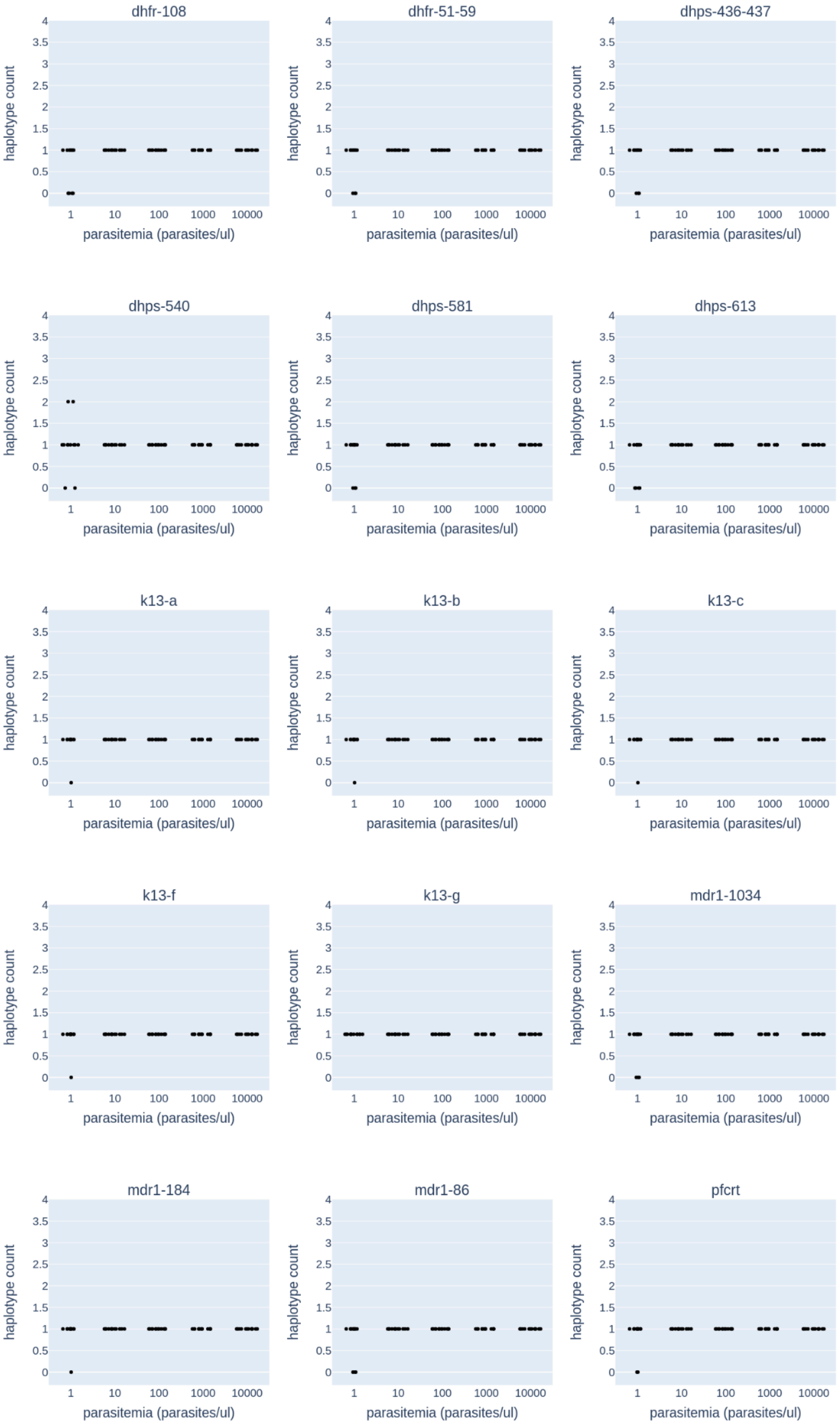

Figure S5. Haplotype counts in monoclonal control samples at different parasitemias for drug resistance amplicons. This figure shows the relationship between parasitemia and the number of haplotypes detected for amplicons containing known resistance loci. There are 12 samples at each parasitemia, and because the samples are monoclonal, the expected number of haplotypes is one.

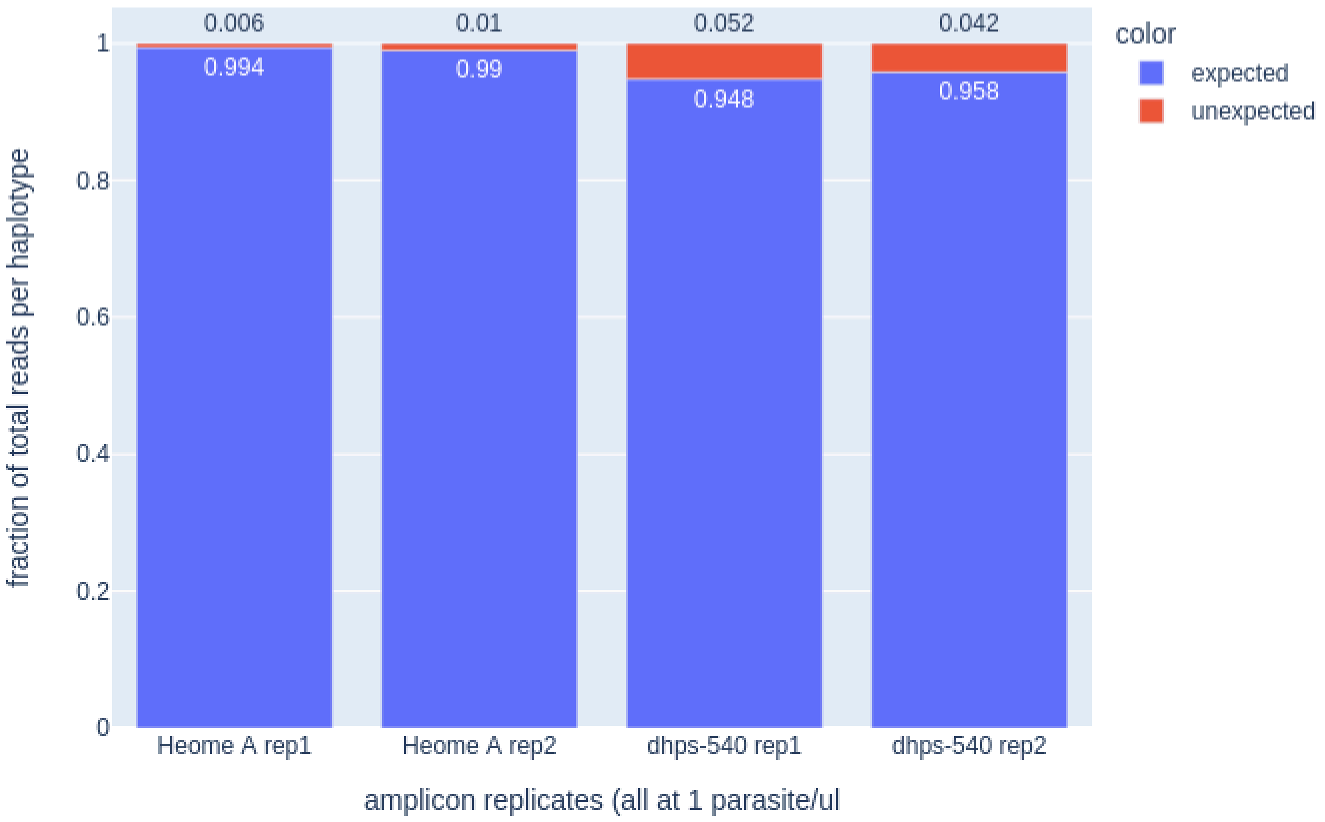

Figure S6. Control samples with unexpected haplotypes. A total of 4 replicate/amplicon/parasitemia combinations (of 1,440 genotyped combinations) had unexpected haplotypes detected (red). All of the replicates with unexpected haplotypes occurred at a concentration of 1 parasite per microliter. The expected haplotype was detected in all samples as the majority variant.

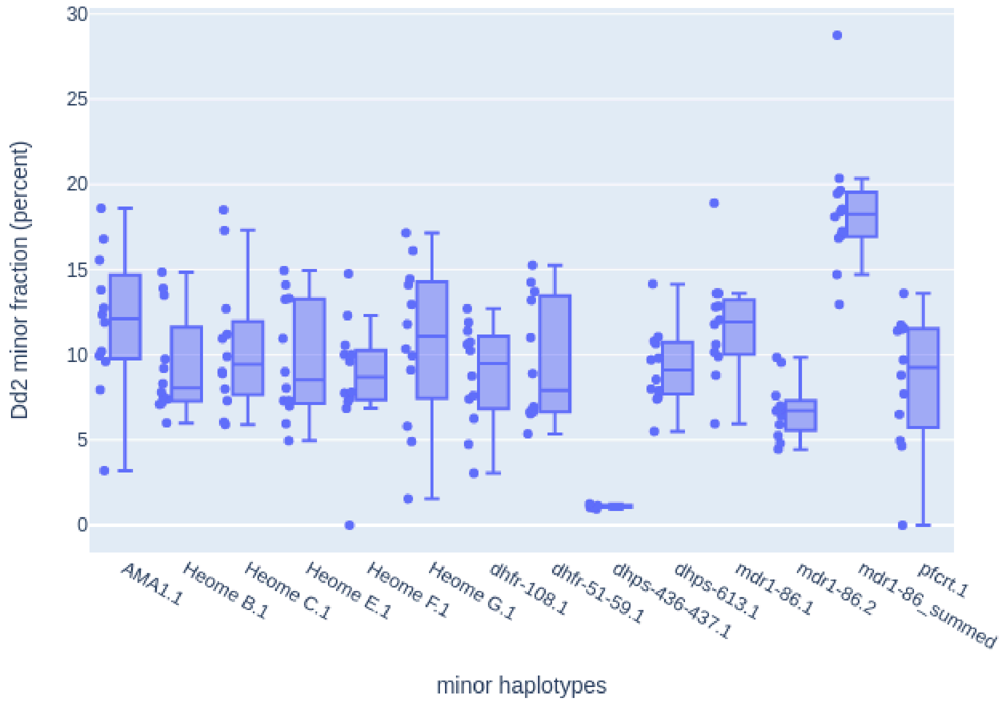

Figure S7. Within sample allele frequency of known mixture of 3D7 and Dd2 (88:12). The amplicon specific within sample allele frequency for the Dd2 minor variant are shown for all 12 samples. Only amplicons that are variable between the two strains are shown. The expected allele frequency is 12 percent. Strain Dd2 has an imperfect second copy of MDR1 which alters the allele frequency at these amplicons (both the frequencies of individual copies and the sum of the frequencies of both copies are shown). At each amplcion, individual points for values are plotted (left) and a box and whiskers plot with maximum and minimum values (right).

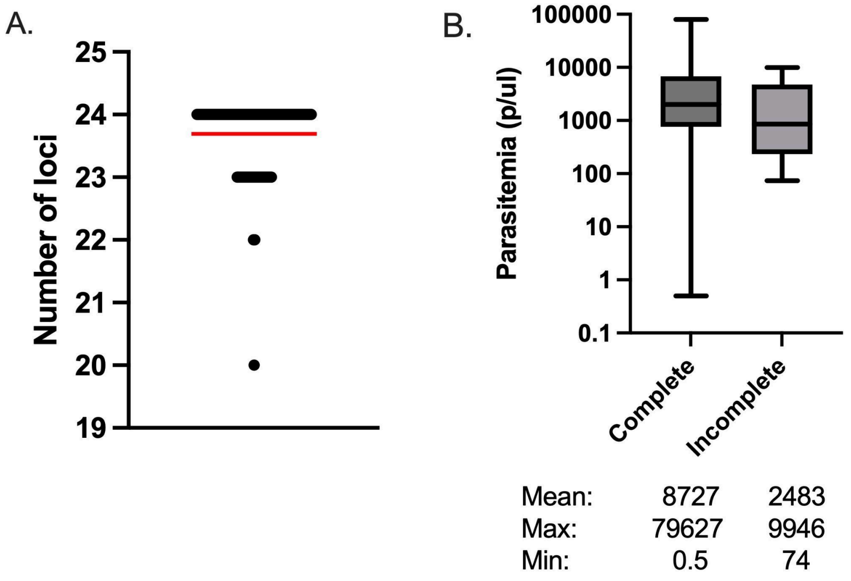

Figure S8. Number of successfully genotyped amplicons per sample among 100 Cameroonian isolates. Panel A shows the number of successful amplicons for each sample, where a successful amplicon is one containing at least one haplotype. The red bar is the mean for all 100 samples. Panel B shows the parasitemia distribution of samples with complete (24 amplicons) versus non-complete (<24 amplicons) genotyping on a log10 scale. The line in the box displays the median parasitemia and the whiskers show the minimum and maximum parasitemia (ns, p= 0.07, t-test).

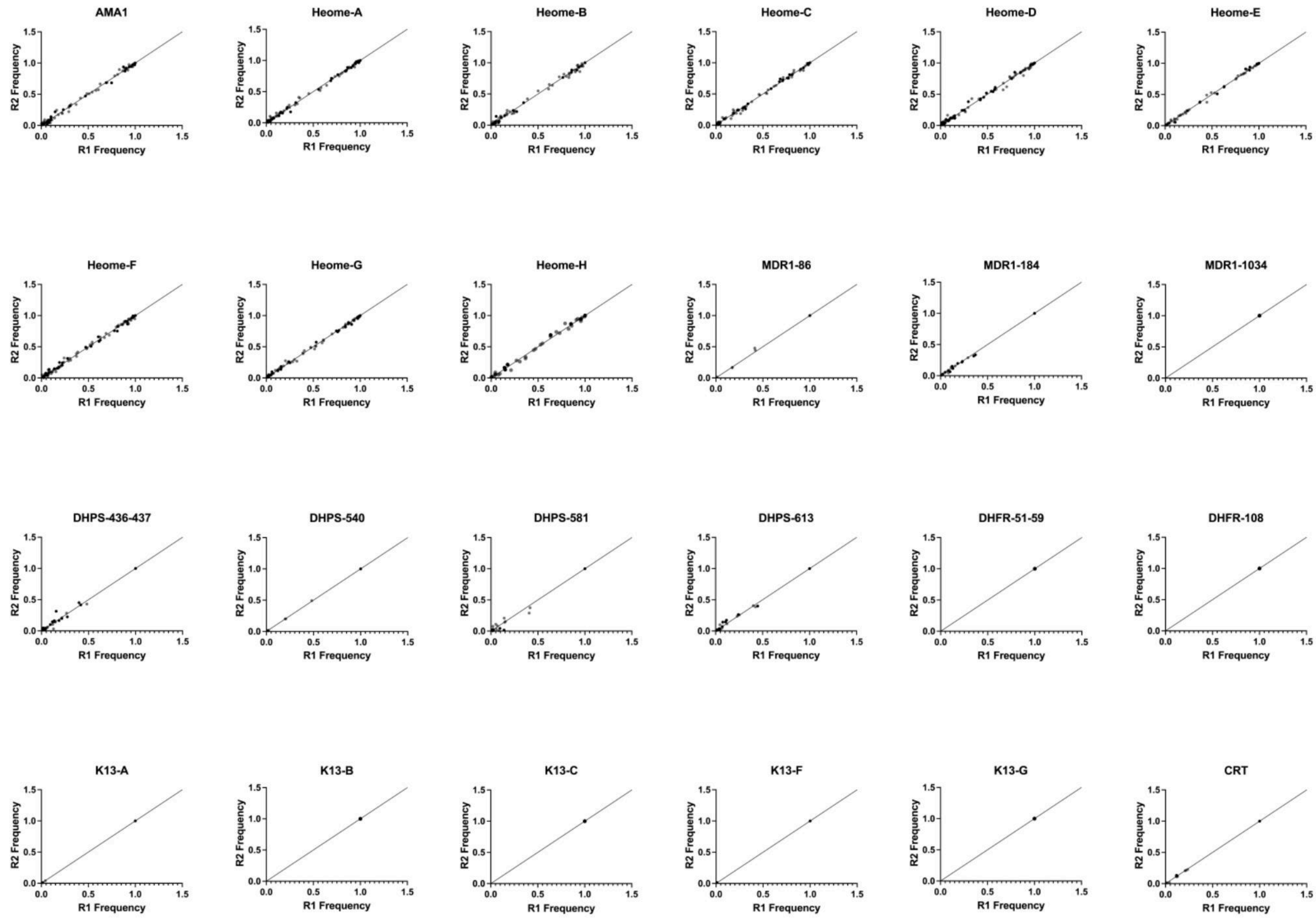
AMA1
Heome-A
Heome-B
Heome-C
Heome-D
Heome-E
Heome-F
Heome-G
Heome-H
MDR1-86
MDR1-184
MDR1-1034
DHPS-436-437
DHPS-540
DHPS-581
DHPS-613
DHFR-51-59
DHFR-108
K13-A
K13-B
K13-C
K13-F
K13-G
CRT
R2 Frequency
R1 Frequency
0.0
0.5
1.0
1.5

Figure S9. Within sample allele frequency comparison of replicates by amplicon for 100 Cameroonian isolates. The calculated within sample haplotype frequency (diversity markers) and antimalarial single nucleotide polymorphisms (SNP) are shown for each replicate. Black dots represent samples with two replicates. For samples where multiple haplotypes were detected for resistance genes, the lowest frequency haplotype was reported.

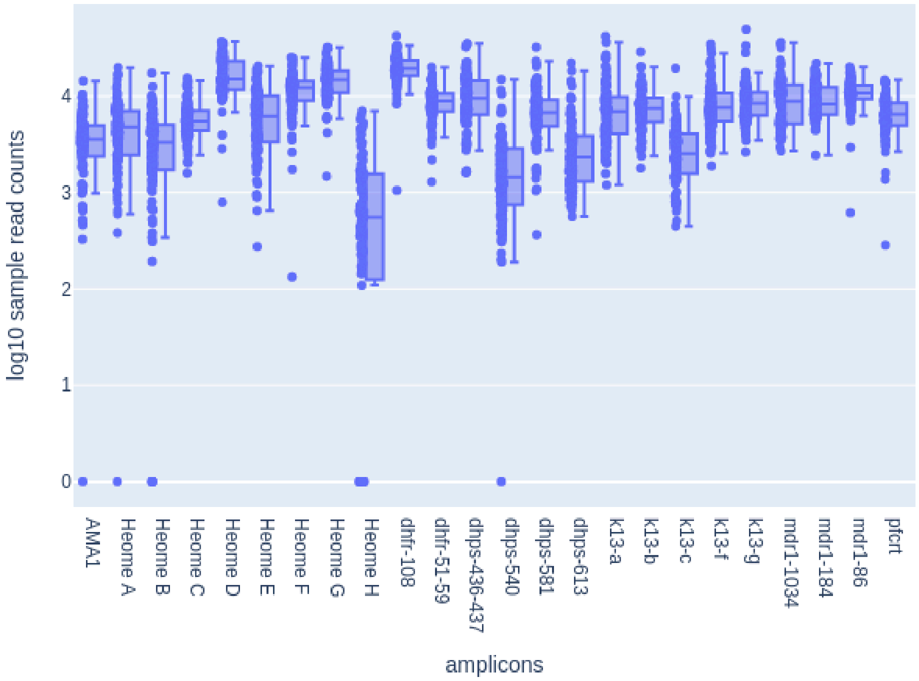

Figure S10. Read depth at each amplicon for 100 Cameroonian isolates. Each amplicon contains 100 Cameroonian isolates. Each isolate contains two replicates which are averaged to yield a single amplicon read count per isolate, illustrated by 100 points per amplicon. At each amplicon, individual points for values are plotted (left) and a box and whiskers plot with maximum and minimum values (right).

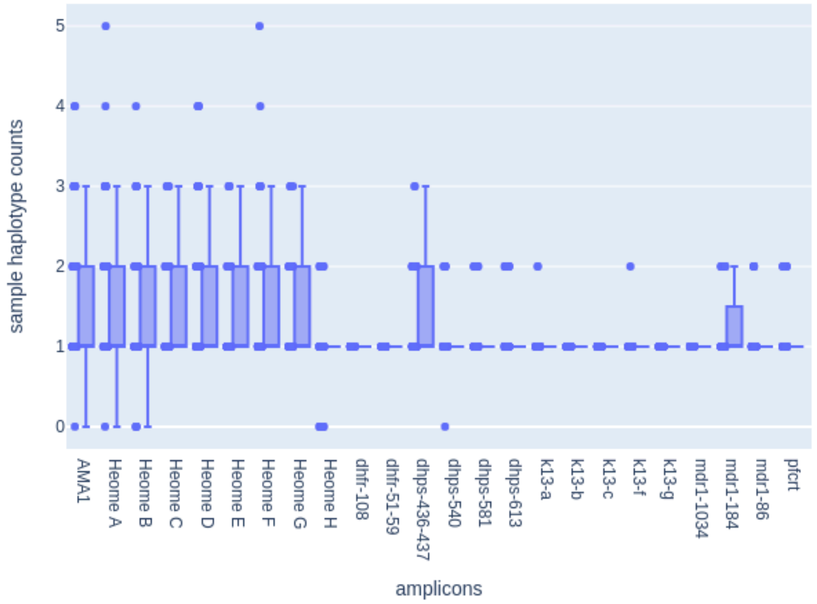

Figure S11. Complexity of infection (COI) at each amplicon for 100 Cameroonian isolates. The COI for each amplicon from the samples is shown. At each amplicon, individual points for values are plotted (left) and a box and whiskers plot with maximum and minimum values (right).

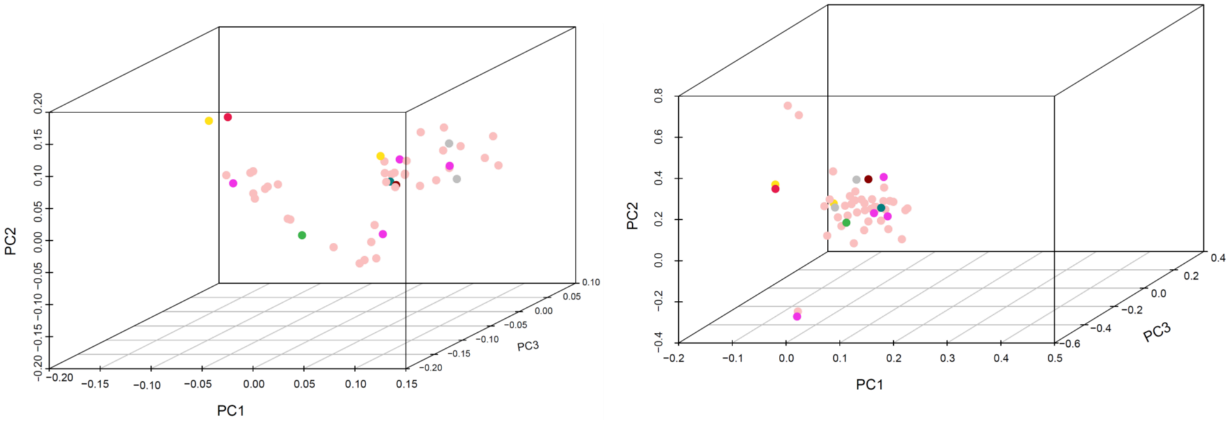

Figure S12. Principal Component Analysis of 50 Cameroonian Samples using *Pf*-SMARRT (A) and Molecular Inversion Probes (MIP)

A

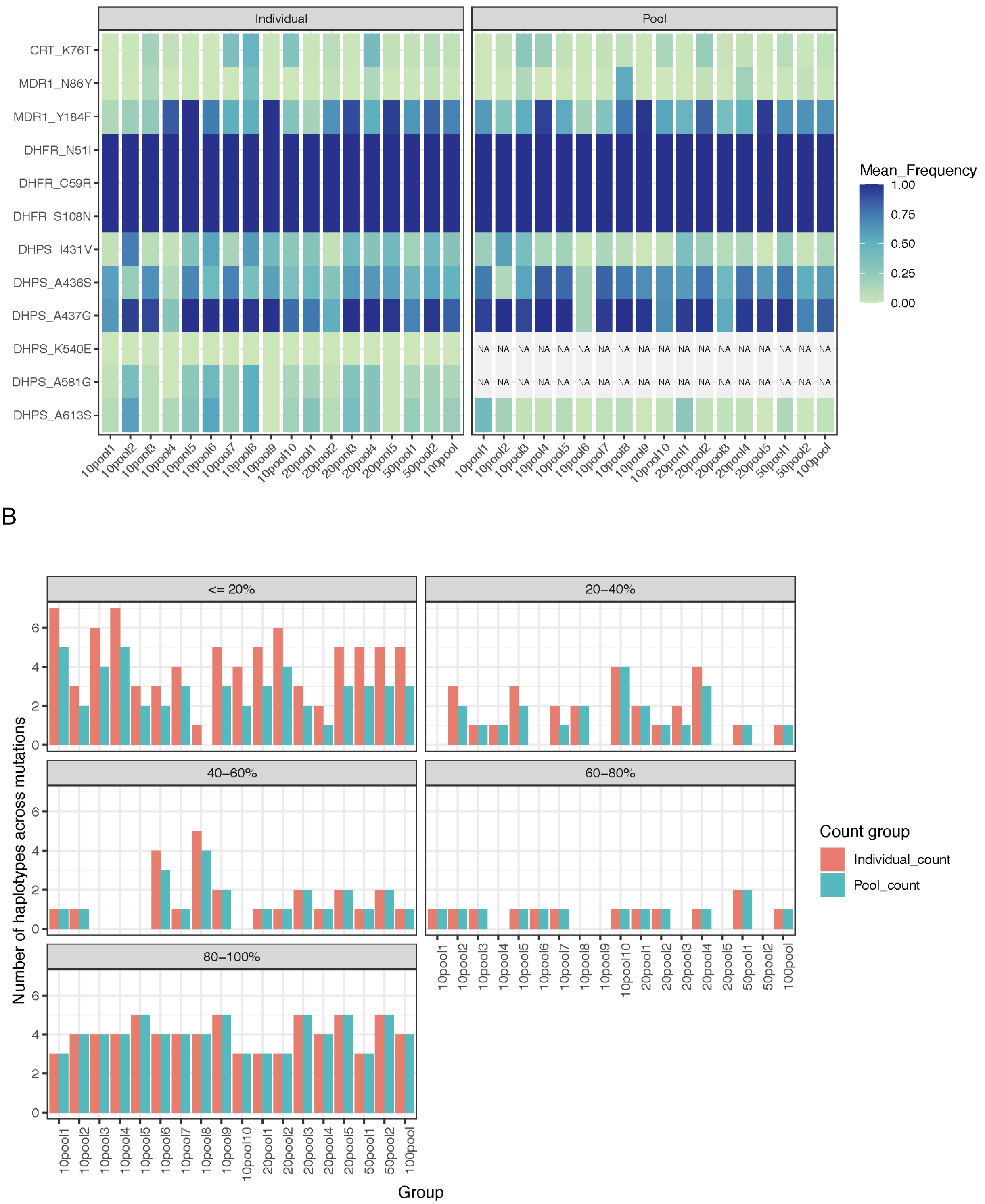

Figure S13. Allele frequency in pooled samples. Weighted mean allele frequency for key resistance mutations shows consistency between pooled and individual samples. Exceptions occur for highly variable and low-frequency variants such as mutations K540E and A581G with frequencies varying from 0.0-0.7% and 0.1-51.5%, respectively, that are not found in pooled samples (A). Lower consistency between the number of total mutations found in pooled versus individual samples can be seen in variants that occur with a frequency lower than 40%, whereas more frequent mutations are successfully identified in both pooled and individual samples (B).

Table S1. Comparison of population characteristics

	Not sequenced (n=130)	Sequenced (n=100)	Total (N=230)	P-value
**Age (years)**	28.0 (21.0, 45.0)	25.0 (19.0, 35.0)	26.0 (20.0, 40.0)	0.28
**Sex?**				0.09
Male	53 (41%)	52 (52%)	105 (46%)	
Female	77 (59%)	48 (48%)	125 (54%)	
**Education**				0.35
Primary	55 (42%)	33 (33%)	88 (38%)	
Secondary	20 (15%)	17 (17%)	37 (16%)	
University	55 (42%)	50 (50%)	105 (46%)	
**Occupation**				0.60
Self-employed	36 (72%)	23 (82%)	59 (76%)	
Employer	5 (10%)	2 (7%)	7 (9%)	
Not working	9 (18%)	3 (11%)	12 (15%)	
**Headache?**				0.72
No	90 (69%)	67 (67%)	157 (68%)	
Yes	40 (31%)	33 (33%)	73 (32%)	
**General fatigue?**				0.56
No	93 (72%)	68 (68%)	161 (70%)	
Yes	37 (28%)	32 (32%)	69 (30%)	
**Vomiting?**				0.59
No	122 (94%)	92 (92%)	214 (93%)	
Yes	8 (6%)	8 (8%)	16 (7%)	
**Cough?**				0.59
No	122 (94%)	92 (92%)	214 (93%)	
Yes	8 (6%)	8 (8%)	16 (7%)	
**Abdominal pain?**				0.19
No	90 (69%)	61 (61%)	151 (66%)	
Yes	40 (31%)	39 (39%)	79 (34%)	
**Gastric pain?**				0.21
No	128 (98%)	100 (100%)	228 (99%)	
Yes	2 (2%)		2 (1%)	
**Other symptoms?**				0.47
No	122 (94%)	96 (96%)	218 (95%)	
Yes	8 (6%)	4 (4%)	12 (5%)	
**Slept under a bednet in the last 24 hours?**				0.11
No	55 (42%)	32 (32%)	87 (38%)	
Yes	75 (58%)	68 (68%)	143 (62%)	
**Nights in the last 7 slept under a bednet?**	7.0 (0.0, 7.0)	7.0 (0.0, 7.0)	7.0 (0.0, 7.0)	0.35
**Number of people in the household?**	2.5 (2.0, 5.0)	3.0 (2.0, 5.0)	3.0 (2.0, 5.0)	0.42
**Number of bednets in the household?**	1.0 (0.0, 2.0)	1.0 (0.0, 2.0)	1.0 (0.0, 2.0)	0.73
**Water within a 2 minutes walk of the household?**				0.17
No	102 (78%)	79 (79%)	181 (79%)	
Yes	21 (16%)	20 (20%)	41 (18%)	
Don't know	7 (5%)	1 (1%)	8 (3%)	0.17
**Type of water source?**				0.28
Stream	3 (2%)	6 (6%)	9 (4%)	
Pond/lake	9 (7%)	7 (7%)	16 (7%)	
Swamp/marsh	9 (7%)	7 (7%)	16 (7%)	
None	102 (78%)	79 (79%)	181 (79%)	
Don't know	7 (5%)	1 (1%)	8 (3%)	
**Traveled in the last 3 months?**				0.34
No	78 (84%)	53 (78%)	131 (81%)	

Yes	15 (16%)	15 (22%)	30 (19%)	
**Is a member of the household working in the forest?**				0.85
No	126 (99%)	97 (99%)	223 (99%)	
Yes	1 (1%)	1 (1%)	2 (1%)	
**Animals in the house?**				0.02
No	120 (94%)	84 (86%)	204 (91%)	
Yes	7 (6%)	14 (14%)	21 (9%)	
**Pregnant?**				0.96
No	122 (96%)	94 (96%)	216 (96%)	
Yes	5 (4%)	4 (4%)	9 (4%)	
**Taken antimalarials in the last 14 days?**				0.63
No	93 (73%)	70 (72%)	163 (73%)	
Yes	28 (22%)	24 (25%)	52 (23%)	
Don't know	4 (3%)	3 (3%)	7 (3%)	
Refused	2 (2%)		2 (1%)	
**Which antimalarials?**				0.51
Quinine	5 (15%)	4 (15%)	9 (15%)	
ACT	16 (47%)	11 (41%)	27 (44%)	
Don't know	11 (32%)	12 (44%)	23 (38%)	
Refused	2 (6%)		2 (3%)	
**Source of antimalarials?**				0.56
Drug seller	6 (22%)	8 (35%)	14 (28%)	
Clinic	14 (52%)	11 (48%)	25 (50%)	
Leftover	7 (26%)	4 (17%)	11 (22%)	
**Lifelong malaria exposure?**				0.34
No	9 (7%)	4 (4%)	13 (6%)	
Yes	118 (93%)	94 (96%)	212 (94%)	
**Number of malaria exposures over lifetime?**	7.0 (3.0, 7.0)	7.0 (6.5, 7.0)	7.0 (5.0, 7.0)	0.51
**Number of malaria exposures in the last year?**	0.0 (0.0, 1.0)	0.0 (0.0, 1.0)	0.0 (0.0, 1.0)	0.62
**Has someone in the household been treated for malaria in the last year?**				0.77
No	14 (11%)	12 (12%)	26 (12%)	
Yes	104 (82%)	76 (78%)	180 (80%)	
Don't know	8 (6%)	8 (8%)	16 (7%)	
Refused	1 (1%)	2 (2%)	3 (1%)	

P-value comparisons across groups for categorical variables are based on chi-square test of homogeneity; p-values for continuous variables are based on Kruskal-Wallis test for median.

Table S2. Within sample allele frequency of known mixture of 3D7 and Dd2 (88:12)^

	AMA1	Heome B	Heome C	Heome E	Heome F	Heome G	DHFR 51/59	DHFR 108	DHPS 436/437*	DHPS 613	MDR1 86 (HAP 1)	MDR1 86 (HAP2)	MDR1 86 (TOTAL)	CRT
Average	0.119	0.094	0.105	0.097	0.087	0.107	0.096	0.088	0.011	0.093	0.118	0.068	0.185	0.085
STDEV	0.042	0.030	0.040	0.035	0.036	0.047	0.036	0.030	0.001	0.023	0.032	0.017	0.038	0.039

*: when detected (5/12 reps)

^: Not all amplicons are variable between the strains. Those with no polymorphisms are not reported.

Table S3. Individual characteristics and sequencing results for 100 participants sequenced with *Pf*-SMARRT

See the uploaded Excel file.

- Tab 1: Individual metadata and averaged frequencies for antimalarial resistance polymorphisms and diversity amplicons in each individual
- Tab 2: Data dictionary for metadata
- Tab 3: Underlying sequencing depth (count) data for sequencing replicates for each individual used to calculate within sample allele frequencies

Table S4. Haplotypes detected for AMA in 100 Cameroonian participants

Haplotype Name	Haplotype Sequence
ama1.00	ATTTGGTAAAGGTATAATTATTGAGAATTCAAAAACTACTTTTTTAACACCGGTAGCTACGGAAAATCAAGATTTAAAAGATGGAGGTTTTGCTTTTCCTCCAACAAATCCTCCTATGTCACCAATGACATTAAATGGTATGAGAGATTTATATAAAAATAATGAATATGTAAAAAATTTAGATGAATTGACTTTA
ama1.01	ATTTGGTAAAGGTATAATTATTGAGAATTCAAATACTACTTTTTTAACACCGGTAGCTACGGAAAATCAAGATTTAAAAGATGGAGGTTTTGCTTTTCCTCCAACAAATCCTCTTATATCACCAATGACATTAGATCATATGAGAGATTCTTATAAAAATAATGAATATGTAAAAAATTTAGATGAATTGACTTTA
ama1.02	ATTTGGTAAAGGTATAATTATTGAGAATTCAAATACTACTTTTTTAAAACCGGTAGCTACGGGAAATCAAGATTTAAAAGATGGAGGTTTTGCTTTTCCTCCAACAAATCCTCTTATATCACCAATGACATTAAATGGTATGAGAGATTTTTATAAAAATAATGAATATGTAAAAAATTTAGATGAATTGACTTTA
ama1.03	ATTTGGTAAAGGTATAATTATTGAGAATTCAAATACTACTTTTTTAACACCGGTAGCTACGGAAAATCAAGATTTAAAAGATGGAGGTTTTGCTTTTCCTCCAACAAAACCTCTTATGTCACCAATGACATTAGATCAAATGAGACATTTTTATAAAGATAATAAATATGTAAAAAATTTAGATGAATTGACTTTA
ama1.04	ATTTGGTAAAGGTATAATTATTGAGAATTCAAATACTACTTTTTTAACACCGGTAGCTACGGGAAATCAATATTTAAAAGATGGAGGTTTTGCTTTTCCTCCAACAGAACCTCATATGTCACCAATGACATTAGATGAAATGAGACATTTTTATAAAGATAATAAATATGTAAAAAATTTAGATGAATTGACTTTA
ama1.05	ATTTGGTAAAGGTATAATTATTGAGAATTCAAATACTACTTTTTTAAAACCGGTAGCTACGGGAAATCAAGATTTAAAAGATGGAGGTTTTGCTTTTCCTCCAACAGAACCTCTTATATCACCAATGACATTAGATGATATGAGAGATTTTTATAAAAATAATGAATATGTAAAAAATTTAGATGAATTGACTTTA
ama1.06	ATTTGGTAAAGGTATAATTATTGAGAATTCAAATACTACTTTTTTAACACCGGTAGCTACGGAAAATCAAGATTTAAAAGATGGAGGTTTTGCTTTTCCTCCAACAGAACCTCTTATGTCACCAATGACATTAGATCAAATGAGACATTTTTATAAAGATAATAAATATGTAAAAAATTTAGATGAATTGACTTTA
ama1.07	ATTTGGTAAAGGTATAATTATTGAGAATTCAAATACTACTTTTTTAACACCGGTAGCTACGGAAAATCAAGATTTAAAAGATGGAGGTTTTGCTTTTCCTCCAACAAAACCTCTTATGTCACCAATGACATTAGATCAAATGAGAGATTTTTATAAAAATAATGAATATGTAAAAAATTTAGATGAATTGACTTTA
ama1.08	ATTTGGTAAAGGTATAATTATTGAGAATTCAAATACTACTTTTTTAACACCGGTAGCTACGGAAAATCAAGATTTAAAAGATGGAGGTTTTGCTTTTCCTCCAACAAAACCTCTTATGTCACCAATGACATTAGATGAAATGAGACATTTTTATAAAGATAATAAATATGTAAAAAATTTAGATGAATTGACTTTA
ama1.09	ATTTGGTAAAGGTATAATTATTGAGAATTCAAATACTACTTTTTTAAAACCGGTAGCTACGGGAAATCAAGATTTAAAAGATGGAGGTTTTGCTTTTCCTCCAACAGAACCTCTTATATCACCAATGACATTAAATGGTATGAGAGATTTTTATAAAAATAATGAATATGTAAAAAATTTAGATGAATTGACTTTA
ama1.10	ATTTGGTAAAGGTATAATTATTGAGAATTCAAATACTACTTTTTTAACACCGGTAGCTACGGGAAATCAATATTTAAAAGATGGAGGTTTTGCTTTTCCTCCAACAGAACCTCTTATGTCACCAATGACATTAGATGAAATGAGACATTTTTATAAAGATAATAAATATGTAAAAAATTTAGATGAATTGACTTTA
ama1.11	ATTTGGTAAAGGTATAATTATTGAGAATTCAAATACTACTTTTTTAACACCGGTAGCTACGGGAAAACAAGATTTAAAAGATGGAGGTTTTGCTTTTCCTCCAACAAATCCTCTTATATCACCAATGACATTAAATGGTATGAAAGATTTTTATAAAGATAATGAAGATGTAAAAAATTTAGATGAATTGACTTTA
ama1.12	ATTTGGTAAAGGTATAATTATTGAGAATTCAAAAACTACTTTTTTAACACCGGTAGCTACGGAAAATCAAGATTTAAAAGATGGAGGTTTTGCTTTTCCTCCAACAAAACCTCTTATGTCACCAATGACATTAGATGATATGAGAGATCTTTATAAAAATAATGAATATGTAAAAAATTTAGATGAATTGACTTTA
ama1.13	ATTTGGTAAAGGTATAATTATTGAGAATTCAAAAACTACTTTTTTAACACCGGTAGCTACGGAAAATCAAGATTTAAAAGATGGAGGTTTTGCTTTTCCTCCAACAGAACCTCTTATGTCACCAATGACATTAGATGATATGAGACGTTTTTATAAAGATAATGAATATGTAAAAAATTTAGATGAATTGACTTTA
ama1.14	ATTTGGTAAAGGTATAATTATTGAGAATTCAAATACTACTTTTTTAACACCGGTAGCTACGGGAAAACAAGATTTAAAAGATGGAGGTTTTGCTTTTCCTCCAACAAATCCTCTTATATCACCAATGACATTAGATCATATGAGAGATTTTTATAAAAAAAATGAATATGTAAAAAATTTAGATGAATTGACTTTA
ama1.15	ATTTGGTAAAGGTATAATTATTGAGAATTCAAATACTACTTTTTTAACACCGGTAGCTACGGGAAATCAAGATTTAAAAGATGGAGGTTTTGCTTTTCCTCCAACAAAACCTCTTATGTCACCAATGACATTAGATGATATGAGACTTTTGTATAAAGATAATGAAGATGTAAAAAATTTAGATGAATTGACTTTA
ama1.16	ATTTGGTAAAGGTATAATTATTGAGAATTCAAAAACTACTTTTTTAACACCGGTAGCTACGGAAAATCAAGATTTAAAAGATGGAGGTTTTGCTTTTCCTCCAACAAAACCTCTTATGTCACCAATGACATTAGATGAAATGAGACATTTTTATAAAGATAATAAATATGTAAAAAATTTAGATGAATTGACTTTA
ama1.17	ATTTGGTAAAGGTATAATTATTGAGAATTCAAATACTACTTTTTTAACACCGGTAGCTACGGAAAATCAAGATTTAAAAGATGGAGGTTTTGCTTTTCCTCCAACAGAACCTCTTATGTCACCAATGACATTAGATCGTATGAGAGATTTTTATAAAAATAATGAAGATGTAAAAAATTTAGATGAATTGACTTTA

ama1.18	ATTTGGTAAAGGTATAATTATTGAGAATTCAAAAACTACTTTTTTAACACCGGTAGCTACGGAAAATCAAGATTTAAAAGATGGAGGTTTTGCTTTTCCTCCAACAGAACCTCTTA<br>TATCACCAATGACATTAGATCAAATGAGACATCTTTATAAAGATAATGAATATGTAAAAAATTTAGATGAATTGACTTTA
ama1.19	ATTTGGTAAAGGTATAATTATTGAGAATTCAAATACTACTTTTTTAACACCGGTAGCTACGGAAAATCAAGATTTAAAAGATGGAGGTTTTGCTTTTCCTCCAACAAATCCTCCTAT<br>GTCACCAATGACATTAGATGATATGAGACTTTTGTATAAAGATAATGAAGATGTAAAAAATTTAGATGAATTGACTTTA
ama1.20	ATTTGGTAAAGGTATAATTATTGAGAATTCAAATACTACTTTTTTAACACCGGTAGCTACGGAAAATCAAGATTTAAAAGATGGAGGTTTTGCTTTTCCTCCAACAAATCCTCCTAT<br>GTCACCAATGACATTAGATCAAATGAGACATTTTTATAAAGATAATAAATATGTAAAAAATTTAGATGAATTGACTTTA
ama1.21	ATTTGGTAAAGGTATAATTATTGAGAATTCAAAAACTACTTTTTTAACACCGGTAGCTACGGAAAATCAAGATTTAAAAGATGGAGGTTTTGCTTTTCCTCCAACAAAACCTCTTAT<br>GTCACCAATGACATTAGATGATATGAGAGATTTTTATAAAGATAATGAATATGTAAAAAATTTAGATGAATTGACTTTA
ama1.22	ATTTGGTAAAGGTATAATTATTGAGAATTCAAATACTACTTTTTTAACACCGGTAGCTACGGAAAATCAAGATTTAAAAGATGGAGGTTTTGCTTTTCCTCCAACAAAACCTCATAT<br>GTCACCAATGACATTAGATCAAATGAGACATTTTTATAAAGATAATAAATATGTAAAAAATTTAGATGAATTGACTTTA
ama1.23	ATTTGGTAAAGGTATAATTATTGAGAATTCAAATACTACTTTTTTAACACCGGTAGCTACGGAAAATCAAGATTTAAAAGATGGAGGTTTTGCTTTTCCTCCAACAAAACCTCTTAT<br>GTCACCAATGACATTAGATCAAATGAGACATTTTTATAAAGATAATGAAGATGTAAAAAATTTAGATGAATTGACTTTA
ama1.24	ATTTGGTAAAGGTATAATTATTGAGAATTCAAATACTACTTTTTTAAAACCGGTAGCTACGGGAAATCAAGATTTAAAAGATGGAGGTTTTGCTTTTCCTCCAACAAATCCTCTTAT<br>ATCACCAATGACATTAAATGGTATGAAAGATTTTTATAAAGATAATGAAGATGTAAAAAATTTAGATGAATTGACTTTA
ama1.25	ATTTGGTAAAGGTATAATTATTGAGAATTCAAATACTACTTTTTTAAAACCGGTAGCTACGGGAAATCAAGATTTAAAAGATGGAGGTTTTGCTTTTCCTCCAACAAATCCTCTTAT<br>ATCACCAATGACATTAAATGGTATGAGAGATTTATATAAAAATAATGAAGATGTAAAAAATTTAGATGAATTGACTTTA
ama1.26	ATTTGGTAAAGGTATAATTATTGAGAATTCAAAAACTACTTTTTTAACACCGGTAGCTACGGAAAATCAAGATTTAAAAGATGGAGGTTTTGCTTTTCCTCCAACAAAACCTCTTAT<br>GTCACCAATGACATTAGATGATATGAGACTTTTGTATAAAGATAATGAAGATGTAAAAAATTTAGATGAATTGACTTTA
ama1.27	ATTTGGTAAAGGTATAATTATTGAGAATTCAAATACTACTTTTTTAACACCGGTAGCTACGGAAAATCAAGATTTAAAAGATGGAGGTTTTGCTTTTCCTCCAACAAAACCTCTTAT<br>GTCACCAATGACATTAGATCAAATGAGACATTTTTATAGAGATAATAAATATGTAAAAAATTTAGATGAATTGACTTTA
ama1.28	ATTTGGTAAAGGTATAATTATTGAGAATTCAAAAACTACTTTTTTAACACCGGTAGCTACGGAAAATCAAGATTTAAAAGATGGAGGTTTTGCTTTTCCTCCAACAGAACCTCTTA<br>TATCACCAATGACATTAGATGATATGAGAGATTCTTATAAAGATAATGAATATGTAAAAAATTTAGATGAATTGACTTTA
ama1.29	ATTTGGTAAAGGTATAATTATTGAGAATTCAAATACTACTTTTTTAAAACCGGTAGCTACGGGAAATCAAGATTTAAAAGATGGAGGTTTTGCTTTTCCTCCAACAAATCCTCTTAT<br>ATCACCAATGACATTAGATCAAATGAGACATTTTTATAAAGATAATGAAGATGTAAAAAATTTAGATGAATTGACTTTA
ama1.30	ATTTGGTAAAGGTATAATTATTGAGAATTCAAAAACTACTTTTTTAACACCGGTAGCTACGGAAAATCAAGATTTAAAAGATGGAGGTTTTGCTTTTCCTCCAACAAATCCTCCTAT<br>GTCACCAATGACATTAGATGATATGAGACTTTTGTATAAAGATAATGAAGATGTAAAAAATTTAGATGAATTGACTTTA
ama1.31	ATTTGGTAAAGGTATAATTATTGAGAATTCAAATACTACTTTTTTAAAACCGGTAGCTACGGAAAATCAAGATTTAAAAGATGGAGGTTTTGCTTTTCCTCCAACAAATCCTCCTAT<br>GTCACCAATGACATTAGATCAAATGAGACATTTTTATAAAGATAATAAATATGTAAAAAATTTAGATGAATTGACTTTA
ama1.32	ATTTGGTAAAGGTATAATTATTGAGAATTCAAATACTACTTTTTTAAAACCGGTAGCTACGGGAAATCAAGATTTAAAAGATGGAGGTTTTGCTTTTCCTCCAACAAATCCTCCTAT<br>GTCACCAATGACATTAAATGGTATGAGAGATTTTTATAAAAATAATGAATATGTAAAAAATTTAGATGAATTGACTTTA
ama1.33	ATTTGGTAAAGGTATAATTATTGAGAATTCAAAAACTACTTTTTTAACACCGGTAGCTACGGAAAATCAAGATTTAAAAGATGGAGGTTTTGCTTTTCCTCCAACAGAACCTCCTA<br>TGTCACCAATGACATTAGATGATATGAGACTTTTGTATAAAGATAATGAAGATGTAAAAAATTTAGATGAATTGACTTTA
ama1.34	ATTTGGTAAAGGTATAATTATTGAGAATTCAAAAACTACTTTTTTAACACCGGTAGCTACGGAAAATCAAGATTTAAAAGATGGAGGTTTTGCTTTTCCTCCAACAGAACCTCTTA<br>TATCACCAATGACATTAGATCAAATGAGAGATTTTTATAAAAATAATGAATATGTAAAAAATTTAGATGAATTGACTTTA
ama1.35	ATTTGGTAAAGGTATAATTATTGAGAATTCAAATACTACTTTTTTAACACCGGTAGCTACGGGAAATCAAGATTTAAAAGATGGAGGTTTTGCTTTTCCTCCAACAAATCCTCCTAT<br>GTCACCAATGACATTAAATGGTATGAGAGATTTATATAAAAATAATGAATATGTAAAAAATTTAGATGAATTGACTTTA
ama1.36	ATTTGGTAAAGGTATAATTATTGAGAATTCAAATACTACTTTTTTAACACCGGTAGCTACGGGAAATCAAGATTTAAAAGATGGAGGTTTTGCTTTTCCTCCAACAAATCCTCCTAT<br>GTCACCAATGACATTAAATGGTATGAGAGATTTTTATAAAAATAATGAATATGTAAAAAATTTAGATGAATTGACTTTA

Table S5. Haplotypes detected for Heome A in 100 Cameroonian participants

Haplotype Name	Haplotype Sequence
heome-a.00	TTATTAATGTCATTTTGTTTTTCTTTATTATTATTATTTTTATTTTTTCCCTGGTTATCTTCCTTATTACCTAGCTTTTTTTTGTTGTCATTATTTTCTGTCTGATTATTATTTTTGACTTT<br>GTTCATCTTTGAATTATCCGTTCTGTTTGTATGTCTCAAGCAAGGTT
heome-a.01	TTATTAATGTCATTTTGTTTTTCTTTATTATTATTATTTTTATTTTTTCCCTGGTTATCTTCCTTATTACCTAGCTTTTTTTTGTTGTCATTATTTTCTGTCTGATTATTATTTTTGACTTT<br>GTTCATCTTTGAATTATTCGTTCTGTTTGTATGTCTCAAGCAAGGTT
heome-a.02	TTATTAATGTCATTTTGTTTTTCTTTATTATTATTATTTTTATTTTTTCCCTGGTTATCTTCCTTATTACCTAGCTTTTTTTTGTTGTCATTATTTTCTGTCTGATTATTATTTTTGACTCT<br>GTTCATCTTTGAATTATCCGTTCTGTTTGTATGTCTCAAGCAAGGTT
heome-a.03	TTATTAATGTCATTTTGTTTTTCTTTATTATTATTATTTTTATTTTTTCCCTGGTTATCTTCCTTATTACCTAGCTTTTTTTTGTTGTCATTATTTTCTGTCTGATTATTATTTTTGACTTT<br>GTTCATCCTTGAATTATCCGTTCTGTTTGTATGTCTCAAGCAAGGTT
heome-a.04	TTATTAATGTCATTTTGTTTTTCTTTATTATTATTATTTTTATTTTTTCCCTGGTTATCTTTCTTATTACCTAGCTTTTTTTTGTTGTCATTATTTTCTGTCTGATTATTATTTTTGACTTTG<br>TTCATCTTTGAATTATCCGTTCTGTTTGTATGTCTCAAGCAAGGTT
heome-a.05	TTATTAATGTCATTTTGTTTTTCTTTATTATTATTATTTTTATTTTTTCCCTGGTTATCTTTCTTATTACCTAGCTTTTTTTTGTTGTCATTATTTTCTGTCTGATTATTATTTTTAACTTTG<br>TTCATCTTTGAATTATGCGTTCTGTTTGTATGTCTCAAGCAAGGTT
heome-a.06	TTATTAATGTCATTTTGTTTTTCTTTATTATTATTATTTTTATTTTTTCCCTGGTTATCTTCCTTATTACCTAGCTTTTTTTTGTTGTCATTATTTTCTGTCTGATTATTATTTTTGACTTT<br>GTTCATCTTTGAATTATGCGTTCTGTTTGTATGTCTCAAGCAAGGTT
heome-a.07	TTATTAATGTCATTTTGTTTTTCTTTATTATTATTATTTTTATTTTTTCCCTGGTTATCTTCCTTATTACCTAGCTTTTTTTTGTTGTCATTATTTTCTGTCTGATTATTATTTTTGACTTTT<br>TTCATCTTTGAATTATCCGTTCTGTTTGTATGTCTCAAGCAAGGTT
heome-a.08	TTATTAATGTCATTTTGTTTTTCTTTATTATTATTATTTTTATTTTTTCCCTGGTTATCTTTCTTATTACCTAGCTTTTTTTTGTTGTCATTATTTTCTGTCTGATTATTATTTTTGACTCT<br>GTTCATCTTTGAATTATCCGTTCTGTTTGTATGTCTCAAGCAAGGTT
heome-a.09	TTATTAATGTCATTTTGTTTTTCTTTATTATTATTATTTTTATTTTTTCCCTGGTTATCTTTCTTATTACCTAGCTTTTTTTTGTTGTCATTATTTTCTGTCTGATTATTATTTTTGACTTTG<br>TTCATCTTTGAATTATTCGTTCTGTTTGTATGTCTCAAGCAAGGTT
heome-a.10	TTATTAATGTCATTTTGTTTTTCTTTATTATTATTATTTTTATTTTTTCCCTGGTTATCTTCCTTATTACCTAGCTTTTTTTTGTTGTCATTATTTTCTGTCTGATTATTATTTTTGACTCT<br>GTTCATCTTTGAATTATTCGTTCTGTTTGTATGTCTCAAGCAAGGTT
heome-a.11	TTATTAATGTCATTTTGTTTTTCTTTATTATTATTATTTTTATTTTTTCCCTGGTTATCTTCCTTATTACCTAGCTTTTTTTTGTTGTCATTATTTTCTGTCTGATTATTATTTTTGACTTT<br>GTTCATCTTTGAATTAACCGTTCTGTTTGTATGTCTCAAGCAAGGTT
heome-a.12	TTATTAATGTCATTTTGTTTTTCTTTATTATTATTATTTTTATTTTTTCCCTGGTTATCTTCCTTATTACCTAGCTTTTTTTTGTTGTCATTATTTTCTGTCTGATTATTATTTTTGACTCT<br>GTTCATCTTTGAATTATGCGTTCTGTTTGTATGTCTCAAGCAAGGTT
heome-a.13	TTATTAATGTCATTTTGTTTTTCTTTATTATTATTATTTTTATTTTTTCCCTGGTTATCTTCCTTATTACCTAGCTTTTTTTTGTTGTCATTATTTTCTGTCTGATTATTATTTTTGACTTT<br>GTTAATCTTTGAATTATCCGTTCTGTTTGTATGTCTCAAGCAAGGTT
heome-a.14	TTATTAATGTCATTTTGTTTTTCTTTATTATTATTATTTTTATTTTTTCCCTGGTTATCTTTCTTATTACCTAGCTTTTTTTTGTTGTCATTATTTTCTGTCTGATTATTATTTTTGACTCT<br>GTTCATCTTTGAATTATTCGTTCTGTTTGTATGTCTCAAGCAAGGTT
heome-a.15	TTATTAATGTCATTTTGTTTTTCTTTATTATTATTATTTTTATTTTTTCCCTGGTTATCTTTCTTATTACCTAGCTTTTTTTTGTTGTCATTATTTTCTGTCTGATTATTATTTTTGACTTTG<br>TTCATCCTTGAATTATCCGTTCTGTTTGTATGTCTCAAGCAAGGTT
heome-a.16	TTATTAATGTCATTTTGTTTTTCTTTATTATTATTATTTTTATTTTTTCCCTGGTTATCTTCCTTATTACCTAGCTTTTTTTTGTTGTCATTATTTTCTGTCTGATTATTATTTTTGACTTTT<br>TTCATCTTTGAATTATCCATTCTGTTTGTATGTCTCAAGCAAGGTT
heome-a.17	TTATTAATGTCATTTTGTTTTTCTTTATTATTATTATTTTTATTTTTTCCCTGGTTATCTTTCTTATTACCTAGCTTTTTTTTGTTGTCATTATTTTCTGTCTGATTATTATTTTTGACTTTG<br>TTCATCTTTGAATTAACCGTTCTGTTTGTATGTCTCAAGCAAGGTT

heome-a.18	TTATTAATGTCATTTTGTTTTTCTTTATTATTATTATTTTTATTTTTTCCCTGGTTATCTTCCTTATTACCTAGCTTTTTTTTGTTGTCATTATTTTCTGTCTGATTATTATTTTTGACTTTGTTCATCTTTGAATTATCCGTTCTGTTTGAATGTCTCAAGCAAGGTT

Table S6. Haplotypes detected for Heome B in 100 Cameroonian participants

Haplotype Name	Haplotype Sequence
heome-b.00	AATGTACTTATCCCCTGATATGATTTTTCCAATATCATCAAAATCAGTAATCTCTAAATCATTTATATGATTTATATGTATAAATTCTTTTATCTTGTTCAAATAATTTCTGTAATAAAATA<br>TATTCTCTTTATCATCATCAATTATTTTCATATAATCC
heome-b.01	AATGTACTTATCCCCTGATATGATTTCTCCAGTATCATCAAAATCAGTAATCTCTAAATCATTTATATGATTTATATGTATAAATTCTTTTATCTTGTTCAAATAATTTCTGTAATAAAATA<br>TATTCTCTTTATCATCATCAATTATTTTCATATAATCC
heome-b.02	AATGTACTTATCCCCTGATATGATTCCTCCAATATCATCAAAATCAGTAATCTCTAAATCATTTATATGATTTATATGTATAAATTCTTTTATCTTGTTCAAATAATTTCTGTAATAAAATA<br>TATTCTCTTTATCATCATCAATTATTTTCATATAATCC
heome-b.03	AATGTACTTATCCCCTGATATGATTGTTCCAATATCATCAAAATCAGTAATCTCTAAATCATTTATATGATTTATATGTATAAATTCTTTTATCTTGTTCAAATAATTTCTGTAATAAAATA<br>TATTCTCTTTATCATCATCAATTATTTTCATATAATCC
heome-b.04	AATGTACTTATCCCCTGATATGATTTCCCCAATATCATCAAAATCAGTAATCTCTAAATCATTTATATGATTTATATGTATAAATTCTTTTATCTTGTTCAAATAATTTCTGTAATAAAATA<br>TATTCTCTTTATCATCATCAATTATTTTCATATAATCC
heome-b.05	AATGTACTTATCCCCTGATATGATTCCTCCAATATCATCAAAATTAGTAATCTCTAAATCATTTATATGATTTATATGTATAAATTCTTTTATCTTGTTCAAATAATTTCTGTAATAAAATA<br>TATTCTCTTTATCATCATCAATTATTTTCATATAATCC
heome-b.06	AATGTACTTATCCCCTGATATGATTTTTCCAATATCATCAAAATTAGTAATCTCTAAATCATTTATATGATTTATATGTATAAATTCTTTTATCTTGTTCAAATAATTTCTGTAATAAAATA<br>TATTCTCTTTATCATCATCAATTATTTTCATATAATCC
heome-b.07	AATGTACTTATCCCCTGATATGATTTCTCCAATATCATCAAAATCAGTAATCTCTAAATCATTTATATGATTTATATGTATAAATTCTTTTATCTTGTTCAAATAATTTCTGTAATAAAATA<br>TATTCTCTTTATCATCATCAATTATTTTCATATAATCC
heome-b.08	AATGTACTTATCCCCTGATATGATTTCTCCATTATCATCAAAATCAGTAATCTCTAAATCATTTATATGATTTATATGTATAAATTCTTTTATCTTGTTCAAATAATTTCTGTAATAAAATA<br>TATTCTCTTTATCATCATCAATTATTTTCATATAATCC
heome-b.09	AATGTACTTATCCCCTGATATGAGTTCTCCAATATCATCAAAATCAGTAATCTCTAAATCATTTATATGATTTATATGTATAAATTCTTTTATCTTGTTCAAATAATTTCTGTAATAAAAT<br>ATATTCTCTTTATCATCATCAATTATTTTCATATAATCC

Table S7. Haplotypes detected for Heome C in 100 Cameroonian participants

Haplotype Name	Haplotype Sequence
heome-c.0	TACCCACGGGACATTTTAATTTATACCTATTGGAACATTTTTTATTAGGTCTTTCTTTATGATTCTTCTTTAAAATAGGTTCATCATTATTTGAGTAAACTAACAGATCATTATCGTTCT<br>CAAAATTATTATAACCATCAATATTTTTATTTCCATATACATCTATAAT
heome-c.1	TACCCACGGGACATTTTAATTTATACCTATTGGAACATTTTTTATTAGGTCTTTCTTTATGATTCTTCTTTAAAATAGGTTCATCATTATTTGAGTAAACTAACAGATTATTATCGTTCT<br>CAAAATTATTATAACCATCAATATTTTTATTTCCATATACATCTATAAT
heome-c.2	TACCCACGGGACATTTTAATTTATACCTATTGGAACATTTTTTATTAGGTCTTTCTTTATGATTCTTCTTTAAAATAGGTTCATCATTATTTGAGTAAACTAACAGATCATTATCGTTCT<br>CAAATTTATTATAACCATCAATATTTTTATTTCCATATACATCTATAAT
heome-c.3	TACCCACGGGACATTTTAATTTATACCTATTGGAACATTTTTTATTAGGTCTTTCTTTATGATTCTTCTTTAAAATAGGTTCATCATTATTTGAGTAAACTAACAGATCATTATGGTTC<br>TCAAAATTATTATAACCATCAATATTTTTATTTCCATATACATCTATAAT
heome-c.4	TACCCACGGGACATTTTAATTTATACCTATTGGAACATTTTTTATTAGGTCTTTCTTTATGATTCTTCTTTAAAATAGGTTCATCATTATTTGAGTAAACTAACAGATCATTATCCTTCT<br>CAAAATTATTATAACCATCAATATTTTTATTTCCATATACATCTATAAT

Table S8. Haplotypes detected for Heome D in 100 Cameroonian participants

Haplotype Name	Haplotype Sequence
heome-d.00	CATGATTCCTCAAAAGTTTAACATTCCACATAGTATCTGTTAAATTGATAGTTTGCAAACAATTATACGTTTGGTTATTTTTATTTTTAAAAAAGGATATATGATCATCTAAAGATTTATTTTTGAGAAAGATTTCCATATATTCATATATATCTTGAATACACCAG
heome-d.01	CATGATTCCTCAAAAGTTTAACATTCCACATAGTATCTGTTAAATTGATAGTTTGCAAACAATTATACGTTTGGTTATTTTTATTTTTAAAAAAGGATATATGATCATCTAAAGATTTATTTTTGAGAAAGATTTCCATATATTCATATATATCATGAATACACCAG
heome-d.02	CATGATTCCTCAAAAGTTTAACATTCCACATAGTATCTGTTAAATTGATAGTTTGCAAACAATTATACGTTTGGTTATTTTTATTTTTAAAAAAGGATATATGATCATCTAAAGATTTATTTTTGAGAAAGATTTCCATATATTCATATATATCTTGGATACACCAG
heome-d.03	CATGATTCCTCAAAAGTTTAACATTCCACATAGTATCTGTTAAATTGATAGTTTGCAAACAATTATACGTTTGGTTATTTTTATTTTTAAAAAAGGATATATGATCATCTAAACATTTATTTTTGAGAAAGATTTCCATATATTCATATATATCTTGAATACACCAG
heome-d.04	CATGATTCCTCAAAAGTTTAACATTCCACATAGTATCTGTTAAATTGATAGTTTGCAAACAATTATACGTTTGGTTATTTTTATTTTTAAAAAAGGATATATGATCATCTAAACATTTATTTTTGAGAAAGATTTCCATATATTCATATATATCTTGGATACACCAG
heome-d.05	CATGATTCCTCAAAAGTTTAACATTCCACATAGTATCTGTTAAATTGATAGTTTGCAAACAATTATACGTTTGGTTATTTTTATTTTTAAAAAAGGATATATGATCATCTAACGATTTATTTTTGAGAAAGATTTCCATATATTCATATATATCTTGAATACACCAG
heome-d.06	CATGATTCCTCAAAAGTTTAACATTCCACATAGTATCTGTTAAATTGATAGTTTGCAAACAATTATACGTTTGGTTATTTTTATTTTTAAAAAAGGATATATGATCATCTAAAGATTTATTTTTGAGAAAGATTTCCATATATTCATATACATCTTGAATACACCAG
heome-d.07	CATGATTCCTCAAAAGTTTAACATTCCACATAGTATCTGTTAAATTGATAGTTTGCAAACAATTATACGTTTGGTTATTTTTATTTTTAAAAAAGGATATATGATCATCTAAAGATTTATTTTTGAGAAAGATTTCCATATATTCATATATATCTTGTATACACCAG
heome-d.08	CATGATTCCTCAAAAGTTTAACATTCCACATAGTATCTGTTAAATTGATAGTTTGCAAACAATTATACGTTTGGTTATTTTTATTTTTAAAAAAGGATATATGATCATCTAAACATTTATTTTTGAGAAAGATTTCCATATATTCATATACATCTTGAATACACCAG
heome-d.09	CATGATTCCTCAAAAGTTTAACATTCCACATAGTATCTGTTAAATTGATAGTTTGCAAACAATTATACGTTTGGTTATTTTTATTTTTAAAAAAGGATATATGATCATCTAAAGATTTATTTTTGAGAAAGATTTCCATATATTCATATATATCGTGAATACACCAG
heome-d.10	CATGATTCCTCAAAAGTTTAACATTCCACATAGTATCTGTTAAATTGATAGTTTGCAAACAATTATACGTTTGGTTATTTTTATTTTTAAAAAAGGATATATGATCATCTAAAAATTTATTTTTGAGAAAGATTTCCATATATTCATATATATCTTGAATACACCAG
heome-d.11	CATGATTCCTCAAAAGTTTAACATTCCACATAGTATCTGTTAAATTGATAGTTTGCAAACAATTATACCTTTGGTTATTTTTATTTTTAAAAAAGGATATATGATCATCTAAAGATTTATTTTTGAGAAAGATTTCCATATATTCATATATATCTTGAATACACCAG
heome-d.12	CATGATTCCTCAAAAGTTTAACATTCCACATAGTATCTGTTAAATTGATAGTTTGCAAACAATTATACGTTTGGTTATTTTTATTTTTGAAAAAGGATATATGATCATCTAAAGATTTATTTTTGAGAAAGATTTCCATATATTCATATATATCTTGAATACACCAG
heome-d.13	CATGATTCCTCAAAAGTTTAACATTCCACATAGTATCTGTTAAATTGATAGTTTGCAAACAATTATACGTTTGGTTATTTTTATTTTTAAAAAAGGATATATCATCATCTAAAGATTTATTTTTGAGAAAGATTTCCATATATTCATATATATCTTGAATACACCAG
heome-d.14	CATGATTCCTCAAAAGTTTAACATTCCACATAGTATCTGTTAAATTGATAGTTTGCAAACAATTATACGTTTGGTTATTTTTATTTTTAAAAAAGGATATATGATCATCTAAAGATTTATTTTTGAAAAAGATTTCCATATATTCATATACATCTTGAATACACCAG
heome-d.15	CATGATTCCTCAAAAGTTTAACATTCCACATAGTATCTGTTAAATTGATAGTTTGCAAACAATTATACCTTTGGTTATTTTTATTTTTAAAAAAGGATATATGATCATCTAAAGATTTATTTTTGAAAAAGATTTCCATATATTCATATATATCTTGAATACACCAG
heome-d.16	CATGATTCCTCAAAAGTTTAACATTCCACATAGTATCTGTTAAATTGATAGTTTGCAAACAATTATACGTTTCGTTATTTTTATTTTTAAAAAAGGATATATGATCATCTAAAGATTTATTTTTGAGAAAGATTTCCATATATTCATATATATCTTGAATACCCCAG
heome-d.17	CATGATTCCTCAAAAGTTTAACATTCCACATAGTATCTGTTAAATTGATAGTTTGCAAACAATTATACGTTTGGTTATTTTTATTTTTGAAAAAGGATATATGATCATCTAAAGATTTATTTTTGAGAAAGATTTCCATATATTCATATATATCATGAATACACCAG

heome-d.18	CATGATTCCTCAAAAGTTTAACATTCCACATAGTATCTGTTAAATTGATAGTTTGCAAACAATTATACGTTTGGTTATTTTTATTTTTAAAAAAGGATATATGATCATCTAAAGATTTAT<br>TTTTAAGAAAGATTTCCATATATTCATATATATCTTGAATACACCAG
heome-d.19	CATGATTCCTCAAAAGTTTAACATTCCACATAGTATCTGTTAAATTGATAGTTTGCAAACAATTATACGTTTGGTTATTTTTATTTTTAAAAAAGGATATATGATCATCTAAAGATTTAT<br>TTTTAAGAAAGATTTCCATATATTCATATATATCATGAATACACCAG
heome-d.20	CATGATTCCTCAAAAGTTTAACATTCCACATAGTATCTGTTAAATTGATAGTTTGCAAACAATTATACGTTTGGTTATTTTTATTTTTGAAAAAGGATATATGATCATCTAAAGATTTAT<br>TTTTGAGAAAGATTTCCATATATTCATATATATCTTGGATACACCAG
heome-d.21	CATGATTCCTCAAAAGTTTAACATTCCACATAGTATCTGTTAAATTGATAGTTTGCAAACAATTATACGTTTGGTTATTTTTATTTTTAAAAAAGGATATATGATCATCTAAACATTTAT<br>TTTTAAGAAAGATTTCCATATATTCATATATATCTTGAATACACCAG
heome-d.22	CATGATTCCTCAAAAGTTTAACATTCCACATAGTATCTGTTAAATTGATAGTTTGCAAACAATTATACGTTTGGTTATTTTTATTTTTAAAAAAGGATATATGATCATCTAAAGATTTAT<br>TTTTCAGAAAGATTTCCATATATTCATATATATCATGAATACACCAG
heome-d.23	CATGATTCCTCAAAAGTTTAACATTCCACATAGTATCTGTTAAATTGATAGTTTGCAAACAATTATACGTTTCGTTATTTTTATTTTTAAAAAAGGATATATGATCATCTAAAGATTTAT<br>TTTTGAGAAAGATTTCCATATATTCATATATATCTTGAATACACCAG
heome-d.24	CATGATTCCTCAAAAGTTTAACATTCCACATAGTATCTGTTAAATTGATAGTTTGTAAACAATTATACGTTTGGTTATTTTTATTTTTAAAAAAGGATATATGATCATCTAAAGATTTAT<br>TTTTGAGAAAGATTTCCATATATTCATATATATCTTGGATACACCAG
heome-d.25	CATGATTCCTCAAAAGTTTAACATTCCACATAGTATCTGTTAAATTGATAGTTTGCAAACAATTATACGTTTCGTTATTTTTATTTTTAAAAAAGGATATATGATCATCTAAAGATTTAT<br>TTTTGAGAAAGATTTCCATATATTCATATATATCTTGGATACACCAG
heome-d.26	CATGATTCCTCAAAAGTTTAACATTCCACATAGTATCTGTTAAATTGATAGTTTGCAAACAATTATACCTTTGGTTATTTTTATTTTTAAAAAAGGATATATGATCATCTAAAGATTTAT<br>TTTTGAGAAAGATTTCCATATATTCATATATATCATGAATACACCAG
heome-d.27	CATGATTCCTCAAAAGTTTAACATTCCACATAGTATCTGTTAAATTGATAGTTTGCAAACAATTATACGCTTGGTTATTTTTATTTTTAAAAAAGGATATATGATCATCTAAAGATTTAT<br>TTTTGAGAAAGATTTCCATATATTCATATATATCTTGGATACACCAG
heome-d.28	CATGATTCCTCAAAAGTTTAACATTCCACATAGTATCTGTTAAATTGATAGTTTGCAAACAATTATACGTTTGGTTATTTTTATTTTTGAAAAAGGATATATGATCATCTAAAGATTTAT<br>TTTTGAGAAAGATTTCCATATATTCATATATATCGTGAATACACCAG

Table S9. Haplotypes detected for Heome E in 100 Cameroonian participants

Haplotype Name	Haplotype Sequence
heome-e.0	GTATCTTTTCCATATATAATAACGCATGAATTCTTTAAATATATCTTTTACTTTATCTGTAAGTTTCATGGAATCTATTATTTCATGTGCATATTTTATTTTTGCCTCGTTTATATATTTAC<br>CTGCTTCTAATTTACATAAACATTTTTCTATTAAGTTTTGA
heome-e.1	GTATCTTTTCCATATATAATAACGCATGAATTCTTTAAATATATCTTTTACTTTATCTGTAAGTTTCATGGAATCTATTATTTCATGTGCATATTTTATTTTTGCCTCGTTTATATATTTAC<br>CTGCTTTTAATTTACATAAACATTTTTCTATTAAGTTTTGA
heome-e.2	GTATCTTTTCCATATATAATAACGCATGAATTCTTTAAATATATCTTTTACTTTATCTGTAAGTTTCATGGAATCTATTATTTCATCTGCATATTTTATTTTTGCCTCGTTTATATATTTAC<br>CTGCTTCTAATTTACATAAACATTTTTCTATTAAGTTTTGA
heome-e.3	GTATCTTTTCCATATATAATAACGCATGAATTCTTTAAATATATCTTTTACTTTATCTGTAAGTTTCATGGAATCTATTATTTCTTCTGCATATTTTATTTTTGCCTCGTTTATATATTTAC<br>CTGCTTCTAATTTACATAAACATTTTTCTATTAAGTTTTGA
heome-e.4	GTATCTTTTCCATATATAATAACGCATGAATTCTTTAAATATATCTTTTACTTTATCTGTAAGTTTCATGGAATCTATTATTTCATCTGCATATTTTATTTTTGCCTCGTTTATATATTTAC<br>CTGCTTTTAATTTACATAAACATTTTTCTATTAAGTTTTGA
heome-e.5	GTATCTTTTCCATATATAATAACGCATGAATTCTTTAAATTTATCTTTTACTTTATCTGTAAGTTTCATGGAATCTATTATTTCATCTGCATATTTTATTTTTGCCTCGTTTATATATTTAC<br>CTGCTTCTAATTTACATAAACATTTTTCTATTAAGTTTTGA
heome-e.6	GTATCTTTTCCATATATAATAACGCATGAATTCTTTAAATATATCTTTTACTTTATCTGTAAGTTTCATGGAATCTATTATTTCTTCTGCATATTTTATTTTTGCCTCGTTTATATATTTAC<br>CTGCTTTTAATTTACATAAACATTTTTCTATTAAGTTTTGA
heome-e.7	GTATCTTTTCCATATATAATAACGCATGAATTCTTTAAATATATCTTTTACTTTATCTGTAAGTTTCATGGAATCTATTATTTCATGTGCATATTTTATTTTTGCCTCGTTTATATATTCAC<br>CTGCTTTTAATTTACATAAACATTTTTCTATTAAGTTTTGA

Table S10. Haplotypes detected for Heome F in 100 Cameroonian participants

Haplotype Name	Haplotype Sequence
heome-f.00	TTTGGGTAGATATCGTTATAAGGGCTATAATGTATTGACTTCTCCGGGTTTGTTACATTATCATTACAACCAATATTATTAAGGTTAATATTATTTTTATCATTATTATTGTTGTTTTCAT<br>TTGTGTGGTTATATAATAACAATGATGTGTCGTCTATTGGATCATTT
heome-f.01	TTTGGGTAGATATCGTTATAAGGGCTATAATGTATTGACTTCTCCGGGTTGGTTACATTATCATTACAACCAATATTATTAAGGTTAATATTATTTTTATCATTATTATTGTTGTTTTCAT<br>TTGTGTGGTTATATAATAACAATGATGTGTCGTCTATTGGATCATTT
heome-f.02	TTTGGGTAGATATCGTTATAAGGGCTATAATGTATTGACTTCTCCGGGTTGGTTACATTATCATTACAACCAATATTATTAAGGTTCATATTATTTTTATCATTATTATTGTTGTTTTCA<br>TTTGTGTGGTTATATAATAACAATGATGTGTCGTCTATTGGATCATTT
heome-f.03	TTTGGGTAGATATCGTTATAAGGGCTATAATGTATTGACTTCTCCGGGTTTGTTACATTATCATTACAACCAATATTATTAAGGTTCATATTATTTTTATCATTATTATTGTTGTTTTCAT<br>TTGTGTGGTTATATAATAACAATGATGTGTCGTCTATTGGATCATTT
heome-f.04	TTTGGGTAGATATCGTTATAAGGGCTATAATGTATTGACTTCTCCGGGTTTGTTACATTATCATTACAACCAATATTATTAAGGTTAATACTATTTTTATCATTATTATTGTTGTTTTCAT<br>TTGTGTGGTTATATAATAACAATGATGTGTCGTCTATTGGATCATTT
heome-f.05	TTTGGGTAGATATCGTTATAAGGGCTATAATGTATTGACTTCTCCGGGTTGGTTACATTATCATTACAACCAATATTATTAAGGTTAATACTATTTTTATCATTATTATTGTTGTTTTCA<br>TTTGTGTGGTTATATAATAACAATGATGTGTCGTCTATTGGATCATTT
heome-f.06	TTTGGGTAGATATCGTTATAAGGGCTATAATGTATTGACTTCTCCGGGTTTGTTACATTATCATTACAACCAATATTATTAAGGTTCATATTATTTTTATCATTATTATTCTTGTTTTCAT<br>TTGTGTGGTTATATAATAACAATGATGTGTCGTCTATTGGATCATTT
heome-f.07	TTTGGGTAGATATCGTTATAAGGGCTATAATGTATTGACTTCTCCGGGTTTGTTACATTATCATTACAACCAATATTATTAAGGTTAATATTATTTTTACCATTATTATTGTTGTTTTCAT<br>TTGTGTGGTTATATAATAACAATGATGTGTCGTCTATTGGATCATTT
heome-f.08	TTTGGGTAGATATCGTTATAAGGGCTATAATGTATTGACTTCTCCGGGTTTGTTACATTATCATTACAACCAATATTATTAAGGTTAATATTATTTTTATCATTATTATTCTTGTTTTCAT<br>TTGTGTGGTTATATAATAACAATGATGTGTCGTCTATTGGATCATTT
heome-f.09	TTTGGGTAGATATCGTTATAAGGGCTATAATGTATTGACTTCTCCGGGTTTGTTACATTATCATTACAACCAATATTATTAAGGTTAATATTATTTTTATCATTATTATTGTTGTTTTCAT<br>TTGTGTGGTTATATAATAACAATGATGTGTCGTCTATTAGATCATTT
heome-f.10	TTTGGGTAGATATCGTTATAAGGGATATAATGTATTGACTTCTCCGGGTTGGTTACATTATCATTACAACCAATATTATTAAGGTTCATATTATTTTTATCATTATTATTGTTGTTTTCAT<br>TTGTGTGGTTATATAATAACAATGATGTGTCGTCTATTGGATCATTT
heome-f.11	TTTGGGTAGATATCGTTATAAGGGCTATAATGTATTGACTTCTCCGGGTTGGTTACATTATCATTACAACCAATATTATTAAGGTTAATATTATTTTTACCATTATTATTGTTGTTTTCA<br>TTTGTGTGGTTATATAATAACAATGATGTGTCGTCTATTGGATCATTT
heome-f.12	TTTGGGTAGATATCGTTATAAGGGCTATAATGTATTGACTTCTCCGGGTTGGTTACATTATCATTACAACCAATATTATTAAGGTTAATATTATTTTTATCATTATTATTCTTGTTTTCAT<br>TTGTGTGGTTATATAATAACAATGATGTGTCGTCTATTGGATCATTT

Table S11. Haplotypes detected for Heome G in 100 Cameroonian participants

Haplotype Name	Haplotype Sequence
heome-g.0	AATGATGTAGATGCTAACACAACATCCTGGTCAGTATCTTGTGATAAATAATTTAATTTGTCCGCTAAAAAATAAAGTATAATTTCACAGCTTTTTTTATTTGTATCTGTGTTTTTCAT<br>GTTATCATAAAAAGACAAAGATGAAGTAGCACATGAGTTAATTAT
heome-g.1	AATGATGTAGATGCTAATAGCACATCCTGGTCAGTATCTTGTGATAAATAATTTAATTTATCCGCTAAAAAATAAAGTATAATTTCACAGCTTTTTTTATTTGTATCTGTGTTTTTCAT<br>GTTATCATAAAAAGACAAAGATGAAGTAGCACATGAGTTAATTAT
heome-g.2	AATGATGTAGATGCTAACACAACATCCTGGTCAGTATCTTGTGATAAATAATTTAATTTATCCGCTAAAAAATAAAGTATAATTTCACAGCTTTTTTTATTTGTATCTGTGTTTTTCAT<br>GTTATCATAAAAAGACAAAGATGAAGTAGCACATGAGTTAATTAT
heome-g.3	AATGATGTAGATGCTAATAGCACATCCTGGTCAGTATCTTGTGATAAATAATTTAATTTGTCCGCTAAAAAATAAAGTATAATTTCACAGCTTTTTTTATTTGTATCTGTGTTTTTCAT<br>GTTATCATAAAAAGACAAAGATGAAGTAGCACATGAGTTAATTAT
heome-g.4	AATGATGTAGATGCTAACACAACATCCTGGTCAGTATCTTGTGATAAATAATTTAATTTGTCCGCTAAAAAATAAAGTATAATTTCACAGCTTTTTTTATTTGTATCTGTGTTTTTCAT<br>GTTATCGTAAAAAGACAAAGATGAAGTAGCACATGAGTTAATTAT
heome-g.5	AATGATGTAGATGCTAACACAACATCCTGGTCAGTATCTTGTGATAAATAATTTAATTTATCCGATAAAAAATAAAGTATAATTTCACAGCTTTTTTTATTTGTATCTGTGTTTTTCAT<br>GTTATCATAAAAAGACAAAGATGAAGTAGCACATGAGTTAATTAT

Table S12. Haplotypes detected for Heome H in 100 Cameroonian participants

Haplotype Name	Haplotype Sequence
heome-h.0	AAGAGTGTTAATAATACTACAGCTAGTAATATTTCCAAATCCAAAAATGAACTTGAAAAAAAGTCATCAGTTAATAATAATGCTTATATTAAAATTATCGAAAATTATGATTTATTATGGTTAAAATTAATAGAAATTGAATTATGTTGTAATAATAAAAATTTAAATCCTATAA
heome-h.1	AAGAGTGTTAATAATACTACAGCTAGTCATATTTCCAAATCCAAAAATGAACTTGAAAAAAAGTCATCAGCTAATAATAATGCTTATATTAAAATAATCGAAAATTATGATTTATTATGGTTAAAATTAATAGAAATTGAATTATGTTGTAATAATAAAAATTTAAATCCTATAA
heome-h.2	AAGAGTGTTAATAATACTACAGCTAGTCATATTTCCAAATCCAAAAATGAACTTGAAAAAAAGTCATCAGCTAATAATAATGCTTATATTAAAATTATCGAAAATTATGATTTATTATGGTTAAAATTAATAGAAATTGAATTATGTTGTAATAATAAAAATTTAAATCCTATAA
heome-h.3	AAGAGTGTTAATAATACTACAGCTAGTCATATTTCCAAATCCAAAAATGAACTTGAAAAAAAGTCATCAGTTAATAATAATGCTTATATTAAAATTATCGAAAATTATGATTTATTATGGTTAAAATTAATAGAAATTGAATTATGTTGTAATAATAAAAATTTAAATCCTATAA
heome-h.4	AAGAGTGTTAATAATACTACAGCTAGTAATATTTCCAAATCCAAAAATGAACTTGAAAAAAAGTCATCAGCTAATAATAATGCTTATATTAAAATAATCGAAAATTATGATTTATTATGGTTAAAATTAATAGAAATTGAATTATGTTGTAATAATAAAAATTTAAATCCTATAA

Table S13. ITrue Index ordering

Primer Name	Primer Sequence
iTru5_01_A	AATGATACGGCGACCACCGAGATCTACACACCGACAAACACTCTTTCCCTA*C
iTru5_01_B	AATGATACGGCGACCACCGAGATCTACACAGTGGCAAACACTCTTTCCCTA*C
iTru5_01_C	AATGATACGGCGACCACCGAGATCTACACCACAGACTACACTCTTTCCCTA*C
iTru5_01_D	AATGATACGGCGACCACCGAGATCTACACCGACACTTACACTCTTTCCCTA*C
iTru5_01_E	AATGATACGGCGACCACCGAGATCTACACGACTTGTGACACTCTTTCCCTA*C
iTru5_01_F	AATGATACGGCGACCACCGAGATCTACACGTGAGACTACACTCTTTCCCTA*C
iTru5_01_G	AATGATACGGCGACCACCGAGATCTACACGTTCCATGACACTCTTTCCCTA*C
iTru5_01_H	AATGATACGGCGACCACCGAGATCTACACTAGCTGAGACACTCTTTCCCTA*C
iTru5_02_A	AATGATACGGCGACCACCGAGATCTACACCTTCGCAAACACTCTTTCCCTA*C
iTru5_02_B	AATGATACGGCGACCACCGAGATCTACACGTGGTATGACACTCTTTCCCTA*C
iTru5_02_C	AATGATACGGCGACCACCGAGATCTACACCACTGTAGACACTCTTTCCCTA*C
iTru5_02_D	AATGATACGGCGACCACCGAGATCTACACAGACGCTAACACTCTTTCCCTA*C
iTru5_02_E	AATGATACGGCGACCACCGAGATCTACACCAACTCCAACACTCTTTCCCTA*C
iTru5_02_F	AATGATACGGCGACCACCGAGATCTACACAACACGCTACACTCTTTCCCTA*C
iTru5_02_G	AATGATACGGCGACCACCGAGATCTACACTGGATGGTACACTCTTTCCCTA*C
iTru5_02_H	AATGATACGGCGACCACCGAGATCTACACTTCGAAGCACACTCTTTCCCTA*C
iTru5_03_A	AATGATACGGCGACCACCGAGATCTACACAACACCACACACTCTTTCCCTA*C
iTru5_03_B	AATGATACGGCGACCACCGAGATCTACACTGAGCTGTACACTCTTTCCCTA*C
iTru5_03_C	AATGATACGGCGACCACCGAGATCTACACCACAGGAAACACTCTTTCCCTA*C
iTru5_03_D	AATGATACGGCGACCACCGAGATCTACACTGACAACCACACTCTTTCCCTA*C
iTru5_03_E	AATGATACGGCGACCACCGAGATCTACACTGTTCCGTACACTCTTTCCCTA*C
iTru5_03_F	AATGATACGGCGACCACCGAGATCTACACCCTAGAGAACACTCTTTCCCTA*C
iTru5_03_G	AATGATACGGCGACCACCGAGATCTACACGCATAACGACACTCTTTCCCTA*C
iTru5_03_H	AATGATACGGCGACCACCGAGATCTACACCAGTGCTTACACTCTTTCCCTA*C
iTru5_04_A	AATGATACGGCGACCACCGAGATCTACACCGTATCTCACACTCTTTCCCTA*C
iTru5_04_B	AATGATACGGCGACCACCGAGATCTACACCGTCAAGAACACTCTTTCCCTA*C
iTru5_04_C	AATGATACGGCGACCACCGAGATCTACACCCATGAACACACTCTTTCCCTA*C
iTru5_04_D	AATGATACGGCGACCACCGAGATCTACACGGTACTTCACACTCTTTCCCTA*C
iTru5_04_E	AATGATACGGCGACCACCGAGATCTACACACCGCTATACACTCTTTCCCTA*C
iTru5_04_F	AATGATACGGCGACCACCGAGATCTACACTTCCAGGTACACTCTTTCCCTA*C
iTru5_04_G	AATGATACGGCGACCACCGAGATCTACACTCGAACCTACACTCTTTCCCTA*C
iTru5_04_H	AATGATACGGCGACCACCGAGATCTACACTAGTGCCAACACTCTTTCCCTA*C
iTru5_05_A	AATGATACGGCGACCACCGAGATCTACACGGTACGAAACACTCTTTCCCTA*C
iTru5_05_B	AATGATACGGCGACCACCGAGATCTACACAAGCATCGACACTCTTTCCCTA*C
iTru5_05_C	AATGATACGGCGACCACCGAGATCTACACGCCAATACACACTCTTTCCCTA*C

iTru5_05_D	AATGATACGGCGACCACCGAGATCTACACCTGTATGCACACTCTTTCCCTA*C
iTru5_05_E	AATGATACGGCGACCACCGAGATCTACACCTTAGGACACACTCTTTCCCTA*C
iTru5_05_F	AATGATACGGCGACCACCGAGATCTACACTCAGCCTTACACTCTTTCCCTA*C
iTru5_05_G	AATGATACGGCGACCACCGAGATCTACACACATGCCAACACTCTTTCCCTA*C
iTru5_05_H	AATGATACGGCGACCACCGAGATCTACACGATGGAGTACACTCTTTCCCTA*C
iTru5_06_A	AATGATACGGCGACCACCGAGATCTACACCGATCGATACACTCTTTCCCTA*C
iTru5_06_B	AATGATACGGCGACCACCGAGATCTACACTACTCCAGACACTCTTTCCCTA*C
iTru5_06_C	AATGATACGGCGACCACCGAGATCTACACAGCTACCAACACTCTTTCCCTA*C
iTru5_06_D	AATGATACGGCGACCACCGAGATCTACACTCGACAAGACACTCTTTCCCTA*C
iTru5_06_E	AATGATACGGCGACCACCGAGATCTACACTATGACCGACACTCTTTCCCTA*C
iTru5_06_F	AATGATACGGCGACCACCGAGATCTACACAGCCAACTACACTCTTTCCCTA*C
iTru5_06_G	AATGATACGGCGACCACCGAGATCTACACGATCTTGCACACTCTTTCCCTA*C
iTru5_06_H	AATGATACGGCGACCACCGAGATCTACACCCTCGTTAACACTCTTTCCCTA*C
iTru7_101_01	CAAGCAGAAGACGGCATACGAGATGGTAACGTGTGACTGGAGTTCA*G
iTru7_101_02	CAAGCAGAAGACGGCATACGAGATCAACACAGGTGACTGGAGTTCA*G
iTru7_101_03	CAAGCAGAAGACGGCATACGAGATACACCTCAGTGACTGGAGTTCA*G
iTru7_101_04	CAAGCAGAAGACGGCATACGAGATCATGGATCGTGACTGGAGTTCA*G
iTru7_101_05	CAAGCAGAAGACGGCATACGAGATTGATAGGCGTGACTGGAGTTCA*G
iTru7_101_06	CAAGCAGAAGACGGCATACGAGATCGGTTGTTGTGACTGGAGTTCA*G
iTru7_101_07	CAAGCAGAAGACGGCATACGAGATCAACGAGTGTGACTGGAGTTCA*G
iTru7_101_08	CAAGCAGAAGACGGCATACGAGATACCATAGGGTGACTGGAGTTCA*G
iTru7_101_09	CAAGCAGAAGACGGCATACGAGATGGTGTACAGTGACTGGAGTTCA*G
iTru7_101_10	CAAGCAGAAGACGGCATACGAGATCAGCATACGTGACTGGAGTTCA*G
iTru7_101_11	CAAGCAGAAGACGGCATACGAGATGGACATCAGTGACTGGAGTTCA*G
iTru7_101_12	CAAGCAGAAGACGGCATACGAGATAGAAGGACGTGACTGGAGTTCA*G
iTru7_102_01	CAAGCAGAAGACGGCATACGAGATCGCCTTATGTGACTGGAGTTCA*G
iTru7_102_02	CAAGCAGAAGACGGCATACGAGATCAGGTAAGGTGACTGGAGTTCA*G
iTru7_102_03	CAAGCAGAAGACGGCATACGAGATTTGCAACGGTGACTGGAGTTCA*G
iTru7_102_04	CAAGCAGAAGACGGCATACGAGATGCTGAATCGTGACTGGAGTTCA*G
iTru7_102_05	CAAGCAGAAGACGGCATACGAGATGAACGTGAGTGACTGGAGTTCA*G
iTru7_102_06	CAAGCAGAAGACGGCATACGAGATAACGCACAGTGACTGGAGTTCA*G
iTru7_102_07	CAAGCAGAAGACGGCATACGAGATCGCAACTAGTGACTGGAGTTCA*G
iTru7_102_08	CAAGCAGAAGACGGCATACGAGATTGGCTCTTGTGACTGGAGTTCA*G
iTru7_102_09	CAAGCAGAAGACGGCATACGAGATTGAGCTGTGTGACTGGAGTTCA*G
iTru7_102_10	CAAGCAGAAGACGGCATACGAGATGCCTTAACGTGACTGGAGTTCA*G
iTru7_102_11	CAAGCAGAAGACGGCATACGAGATTGTGGCTTGTGACTGGAGTTCA*G
iTru7_102_12	CAAGCAGAAGACGGCATACGAGATAACCGTGTGTGACTGGAGTTCA*G

iTru7_103_01	CAAGCAGAAGACGGCATACGAGATAATCGCTGGTGACTGGAGTTCA*G
iTru7_103_02	CAAGCAGAAGACGGCATACGAGATGGTCACTAGTGACTGGAGTTCA*G
iTru7_103_03	CAAGCAGAAGACGGCATACGAGATTAGTCTCGGTGACTGGAGTTCA*G
iTru7_103_04	CAAGCAGAAGACGGCATACGAGATACCATGTCGTGACTGGAGTTCA*G
iTru7_103_05	CAAGCAGAAGACGGCATACGAGATAGACATGCGTGACTGGAGTTCA*G
iTru7_103_06	CAAGCAGAAGACGGCATACGAGATGATGGAGTGTGACTGGAGTTCA*G
iTru7_103_07	CAAGCAGAAGACGGCATACGAGATCAGTCACAGTGACTGGAGTTCA*G
iTru7_103_08	CAAGCAGAAGACGGCATACGAGATGTTCTTCGGTGACTGGAGTTCA*G
iTru7_103_09	CAAGCAGAAGACGGCATACGAGATAAGACACCGTGACTGGAGTTCA*G
iTru7_103_10	CAAGCAGAAGACGGCATACGAGATGCCTTCTTGTGACTGGAGTTCA*G
iTru7_103_11	CAAGCAGAAGACGGCATACGAGATTCGAACCTGTGACTGGAGTTCA*G
iTru7_103_12	CAAGCAGAAGACGGCATACGAGATGGAACATGGTGACTGGAGTTCA*G
iTru7_104_01	CAAGCAGAAGACGGCATACGAGATTATGGCACGTGACTGGAGTTCA*G
iTru7_104_02	CAAGCAGAAGACGGCATACGAGATCTACAAGGGTGACTGGAGTTCA*G
iTru7_104_03	CAAGCAGAAGACGGCATACGAGATAATCCAGCGTGACTGGAGTTCA*G
iTru7_104_04	CAAGCAGAAGACGGCATACGAGATCCTCGTTAGTGACTGGAGTTCA*G
iTru7_104_05	CAAGCAGAAGACGGCATACGAGATGCAACCATGTGACTGGAGTTCA*G
iTru7_104_06	CAAGCAGAAGACGGCATACGAGATGGTATAGGGTGACTGGAGTTCA*G
iTru7_104_07	CAAGCAGAAGACGGCATACGAGATCGACCTAAGTGACTGGAGTTCA*G
iTru7_104_08	CAAGCAGAAGACGGCATACGAGATGATCTTGCGTGACTGGAGTTCA*G
iTru7_104_09	CAAGCAGAAGACGGCATACGAGATAAGGCTCTGTGACTGGAGTTCA*G
iTru7_104_10	CAAGCAGAAGACGGCATACGAGATTCCATTGCGTGACTGGAGTTCA*G
iTru7_104_11	CAAGCAGAAGACGGCATACGAGATTACTCCAGGTGACTGGAGTTCA*G
iTru7_104_12	CAAGCAGAAGACGGCATACGAGATCGATGTTCGTGACTGGAGTTCA*G

Supplementary Data Sheet 1. Protocol 1. *Pf*-SMARRT Amplification

Plasmodium falciparum Simultaneous Multiplex Antimalarial Resistance and Relatedness Testing v1.9

Infectious Disease Epidemiology and Ecology Lab University of North Carolina at Chapel Hill, Chapel Hill, NC, USA
www.med.unc.edu/infdis/ideel
References: PMID:29246158, PMID:15132750, PMID:15273102, https://www.ncbi.nlm.nih.gov/pmc/articles/PMC11370457/

Introduction: This is a multiplex PCR protocol for simultaneously amplifying *AMA1*, *Heome*, *CRT*, *MDR1*, *DHFR* and *K13 from Chelex-extracted Dried Blood Spot (DBS) samples*.

The PCR primers were adopted from:
- AMA1 - PMID:29246158
- CRT - designed for this protocol
- MDR1 - PMID:15132750
    - MDR184 designed for this protocol
- DHFR & DHPS - PMID:15273102
- K13 – designed for this protocol
- HeOME: https://www.ncbi.nlm.nih.gov/pmc/articles/PMC11370457/

1. **Materials:**
    1.1 List of primers and expected amplicon lengths based on lab-cultured 3D7 isolate.

Gene	Primer Name	Primer Sequence (5'-3')
AMA1	AMA1_F	CCATCAGGGAAATGTCCAGT
	AMA1_R	TTTCCTGCATGTCTTGAACA
CRT	PFCRT_F_v2	GGTGGAGGTTCTTGTCTTGG
	PFCRT_R_v2	AGTTGTGAGTTTCGGATGTTACA
MDR1	MDR1_86_F	TGTATGTGCTGTATTATCAGGAGGAAC
	MDR1_86_R	AATTGTACTAAACCTATAGATACTAATGATAATATTATAGG
	MDR1_184_F_v2	GTGAGTTCAGGAATTGGTACGA
	MDR1_184_R_v2	GCCTCTTCTATAATGGACATGGT
	MDR1_1034_F	AAAAAGAAGAATTATTGTAAATGCAGCTT
	MDR1_1034_R	GGATCCAAACCAATAGGCAAAA
DHFR	DHFR_51_59_F	TGAGGTTTTTAATAACTACACATTTAGAGGTCT
	DHFR_51_59_R	TATCATTTACATTATCCACAGTTTCTTTGTT
	DHFR_108_F	TGGATAATGTAAATGATATGCCTAATTCTAA
	DHFR_108_R	AATCTTCTTTTTTTAAGGTTCTAGACAATATAACA
DHPS	DHPS_437_F	TGAAATGATAAATGAAGGTGCTAGTGT
	DHPS_437_R	AATACAGGTACTACTAAATCTCTTTCACTAATTTTT
	DHPS_540_F	AATGCATAAAAGAGGAAATCCACAT
	DHPS_540_R	TCGCAAATCCTAATCCAATATCAA
	DHPS_581_F	CCTCGTTATAGGATACTATTTGATATTGGAT
	DHPS_581_R	TGGGCAATAAATCTTTTTCTTGAATA
	DHPS_613_F	TGGATTAGGATTTGCGAAGAAAC
	DHPS_613_R	GTTGTGTATTTATTACAACATTTTGATCATTC
K13	K13-A-432-466_F	GAAAGTGAAGCCTTGTTGAAAGAAG
	K13-A-432-466_R	GTACACATACGCCAGCATTGTTG
	K13-B-461-531_F2	TGATGGTGTAGAATATTTAAATTCGATG
	K13-B-461-531_R	CTACCATTTGACGTAACACCACA

	K13-C-513-570_F	TTTGAAACTGAGGTGTATGATCG
	K13-C-513-570_R	GCTGATGATCTAGGGGTATTCAA
	K13-F-567-630_F	TGGCACCTTTGAATACCCCT
	K13-F-567-630_R	AGGTAATTAAAAGCTGCTCCTGA
	K13-G-660-709_F	TGGCAATTTCTAAATGGTGTACC
	K13-G-660-709_R	GCCAAGCTGCCATTCATTTG
Heome	HeOME-A F	TTTTAGTTTCGGTATTTTGTGTTCCTCTT
	HeOME-A R	AAGAAATTTATCAGAGTTACAAAAGGGAAATC
	HeOME-B F	TTTATCCTTATCATTATTTCCATCATTTTCTGG
	HeOME-B R	AAAAATAAAAGGAACAATGTAATGGTTGAAAA
	HeOME-C F	TCCCGAAAACATATCACTAGATCCAT
	HeOME-C R	AAAAATTAAACATGATGCCACATTTTAGTAGT
	HeOME-D F	AGCTATCATTACATGCTGACACAATAT
	HeOME-D R	GATTAGTTGTGGAGATGATAAAACTATCAAATT
	HeOME-E F	AATTTTCTTATATAACCTAAGTTGATGACTTGG
	HeOME-E R	AGAACAGATGAAGTAACTACTCGATTAAATGA
	HeOME-F F	ATCTTTTTCGTTGTATGTGCATAATCA
	HeOME-F R	AAACATAATTCTAATGATATTGACCTTGTGCA
	HeOME-G F	ACATTCACACAAATAGAAAAATCTTCATTTTTC
	HeOME-G R	ATCAATAATCAAAATCATGATAACAACCAATTT
	HeOME-H F	AATAACTTAAATAAAAATATGGACGGCTCC
	HeOME-H R	GACATTCTTTCAATGCTTCCGAAA

	Chr	fullStart	fullStop	Size	He
**AMA1**	11	1294287	1294523	236	
**DHFR-108**	4	748343	748493	150	
**DHFR-51-59**	4	748173	748361	188	
**DHPS-436-437**	8	549631	549742	111	
**DHPS-540**	8	549958	550119	161	
**DHPS-581**	8	550076	550216	140	
**DHPS-613**	8	550102	550254	152	
**Heome-A**	4	115454	115684	230	0.842943
**Heome-B**	5	213026	213251	225	0.837785
**Heome-C**	14	771406	771633	227	0.789254
**Heome-D**	13	2545064	2545291	227	0.77364
**Heome-E**	8	1344617	1344845	228	0.715463
**Heome-F**	11	1816151	1816378	227	0.703947
**Heome-G**	13	815861	816091	230	0.607549
**Heome-H**	11	408542	408771	229	0.605662
**K13-A**	13	1725576	1725728	152	
**K13-B**	13	1725381	1725645	264	
**K13-C**	13	1725264	1725482	218	
**K13-F**	13	1725105	1725295	190	
**K13-G**	13	1724870	1725020	150	
**MDR1-1034**	5	960948	961039	91	
**MDR1-184**	5	958372	958599	227	
**MDR1-86**	5	958071	958206	135	
**CRT**	7	403503	403693	190	

1.2 Primer pool ratios

- Mix the primers above ***(both forward and reverse)*** into pools 1 and 2 in the volumes below.

Primer	Pool 1 Volume	Primer	Pool 2 Volume
AMA1	20	PFCRT_v2	12
MDR1-86	20	MDR1-184_v2	12
MDR1-1034	15	K13-B	18
K13-A	20	K13-F	12
K13-C	25	DHPS-540	24
K13-G	15	DHPS-613	18
DHPS-436-437	15	DHFR-51-59	24
DHPS-581	15	Heome-A	24
DHFR-108	25	Heome-C	18
Heome-B	25	Heome-E	18
Heome-D	20	Heome-G	36
Heome-F	15	Heome-H	12
**Total Pool 1 Volume**	**460**µL	**Total Pool 2 Volume**	**456**µL

1.3 Other reagents

- Nuclease-free water
- QIAGEN Multiplex PCR kit (Cat.206145 or Cat.206143)
- **ZR-96 DNA Clean & Concentrator-5 (Cat.4024**)
- Qubit or PicoGreen supplies for quantifying product (Thermo Fisher, Cat.Q32854 or Cat.Q33232)
- IDEEL iTru library preparation protocol

1.4 Consumables/Supplies

- Nuclease-free PCR microtubes/strips/plates.
- Nuclease-free 1.5uL Eppendorf tubes (for master mixes).
- Cold plates for microtubes and 1.5uL tubes.
- Consumables/Supplies

1.5 Experiment Setup

- Thaw reagents and samples' DNA on ice.
- Clean pipettes and working bench tops with 70% ethanol
- UV-treat the working surface, all equipment, and tubes/strips.
- Briefly vortex and centrifuge each reagent before use.

2. Methods:

- Dilute the 100µM stock primers down to 10µM working solutions for each forward and reverse primer.
- Prepare the two primer pools as indicated in **section 1.1** by mixing all forward and reverse primers in the ratios shown in **section 1.2.**
- Prepare the PCR master mix based on the table below. Since there are two primer pools, the recipe below applies to both pools 1 and 2. Additionally, two or more PCR replicates are required per primer pool to resolve PCR and sequencing errors. The resulting setup has four PCR reactions per sample, i.e., two PCR replicates for primer pool 1 and two PCR replicates for primer pool 2.

Reagents for a 25µl reaction	1x Reaction
Qiagen PCR Master Mix	12.5 µL
Primer Pool Mix⁺	2.5 µL
Template DNA*	2.0 µL
PCR grade water	8.0 µL
Total Volume:	25.0 µL

⁺The "primer pool mix" represents one for pool 1 that is separate from pool 2

*Template DNA volume can be adjusted depending on the concentration of DNA

- For a sample called ***"001"***, a simple naming convention across the two PCR replicates can be:
  - **001-p1-A** & **001-p1-B** - representing the two replicates for primer pool 1.
  - **001-p2-A** & **001-p2-B** - representing the two replicates for primer pool 2.
- Combine the master mix and sample DNA, seal caps/strips/plates, and place in a thermocycler using the following conditions:

Step		Temperature	Duration
Initial activation step		95°C	15 min
	Denaturation	***94°C***	***30 sec***
**45 Cycles**	***Annealing***	***60°C***	***90 sec***
	Extension	***72°C***	***90 sec***
Final Extension		72°C	10 min

- Spot check PCR products from both pools by gel electrophoresis against a 50 bp or 100 bp ladder, using a 2% agarose gel. Expected amplicon sizes are listed above.

3. **PCR purification**

- During the optimisation of this assay, the **ZR-96 DNA Clean & Concentrator-5 (Cat.4024**) was used. Refer to insert protocol for additional guidance.

4. **Product quantification and mixing of P1 and P2**

- If desired, assay the purified products using capillary electrophoresis to ensure that you have the desired PCR fragments (*approx.* 100-300bp) before proceeding to library preparation.
- For small numbers of samples, quantify the DNA using Qubit reagent and mix equal amounts of P1 and P2 product prior to library preparation.
- For larger numbers of samples, quantification by FI on a plate reader is recommended.
  - Make a dilution series of 6 controls containing DNA in TE-Buffer (0.1ng to 100ng)
  - Dilute PicoGreen reagent 1:200 according to manufacturer's protocol in TE buffer.
  - Mix 48µL diluted PicoGreen with 2 µL DNA or control in a black flat-well microplate.
  - Shimmy plate *gently* against palm to level the fluid in the bottom of the well. Cover plate if needed with optical film.
  - Incubate plate for 2 minutes at room temperature in the multimode plate reader before quantifying the fluorescence signal with 485Ex - 535Em filters.
- Use calculated concentration to mix equal ng of P1 and P2 reaction products for each sample replicate to go into library preparation

5. **Library preparation**

- Refer to the IDEEL iTru library preparation protocol.

Supplementary Data Sheet 2. Protocol 2. Library Preparation

iTru Protocol Adapted from Published Materials

Infectious Disease Epidemiology and Ecology Lab University of North Carolina at Chapel Hill, Chapel Hill, NC, USA
www.med.unc.edu/infdis/ideel
Reference: https://peerj.com/articles/7755/#supplemental-information

1.1 Overview:

This protocol is intended for the preparation of libraries from purified *Pf*-SMARRTT amplicons. The original iTru protocol followed methods for Kapa Hyper Prep Kits using half volumes. This protocol halves the volume again to give ¼ reactions . We're using NEB Modules instead of the actual Kapa Reagents referenced in Adapterama, but the same "Sera-Pure" SpeedBeads. We also omitted the dual size selection. The recipe for SpeedBead Mix is within the supplementary material of this paper.

1.2 Consumables

a. P20 Pipette Tips
b. P200 Pipette Tips
c. Reagent Reservoir (sterile)
d. 96-well PCR plates
e. 50mL Conical Tube (sterile)
f. Foil plate seals
g. Optical plate seals

1.3 Reagents

Library Preparation

a. New England Biotechnology Modules:
    i. End Repair (NEB, Cat.E6050L)
    ii. dA Tailing (NEB, Cat.E6053L)
    iii. T4 Quick DNA Ligase (NEB, Cat.E6056L)
b. 100% Anhydrous Ethanol
c. Molecular Grade Water
d. 1X TE Buffer
e. Elution Buffer (Qiagen, Cat.19086)
f. SpeedBead Mix (see Reference)
g. KAPA HiFi HotStart ReadyMix 2X (KAPA Biosystems, Cat. KK2602)
h. 5uM Stubby iTru Adapter (see Reference)
i. i5 Index Primer Set (**Table S14**)
j. i7 Index Primer Set (**Table S14**)

Library Quantification

Qubit HS dsDNA Kit (Thermo Fisher, Cat.Q32854), *or*
Quant-iT PicoGreen (Thermo Fisher, Cat.Q33232)

1.4 Other Supplies

DynaMag 96-well Magnetic Plate (Thermo Fisher, 12331D)

Multi-mode Plate Reader (FI Unit)
Thermocycler
P10, P20 and P200 12-tip multi-channel pipettor

2.0 Protocol

Notes:

I. Begin from amplified, purified, and combined equimolar *Pf*-SMARRT pools for each sample replicate.
II. Allow SpeedBeads to equalize to room temperature for 30 minutes before using.
III. Keep all reagents and Master Mixes in a cold block or on ice.
IV. Do not vortex NEB enzymes. Gently flick to mix, then spin briefly.
V. Combine Master Mixes immediately before use and return reagents to the freezer promptly.
VI. A list of iTru i5 and i7 Dual Indexing Primers, as well as instructions for synthesizing Stubby Y-yoke Adapters, can be found in the original Adapterama paper within their published Supplemental Materials or an extended list.

2.1 End Repair

1. Insert 12.5μL of purified amplicons in 5 μL of End Repair Master Mix.

    a. 2.5μL buffer, 1.25μL enzyme, & 1.25μL water (1/4 NEB module recommendations).

2. Cover with foil seal, spin plate briefly, then Incubate in a Thermocycler at 20°C for 30 minutes.

3. Add 50μL of SpeedBeads to the 17.5μL volume, mix by gently pipetting, and incubate at room temperature for 5-15 minutes.
    - Magnet capture (DynaMag 96 well magnet plate).
    - Wash twice with 80μL of 80% EtOH.

2.2 A-Tailing

4. Aspirate final wash with 200μL pipette tip, then re-aspirate final ethanol drop in bottom of well with 20μL tip.

If working in 96 well plates, and ethanol is aspirated entirely, the first row or column is likely dry enough for immediate addition of A-tailing Master Mix. If beads appear cracked, they have been allowed to dry for too long.

5. To dry beads, add 12.5μL A-tailing Master Mix.

    a. 1.25μL buffer, 0.75μL enzyme*, & 10.5μL water.

6. Incubate in a Thermocycler at 30°C for 30 minutes.

7. Add 22.5μL of PEG/NaCl to 12.5μL volume, mix by pipetting, and incubate at room temperature for 5-15 minutes. This step re-binds DNA to beads.
    - Magnet capture & wash twice with 80μL of 80% EtOH.

2.3 Stubby Adapter Ligation

8. To dry beads add 1.25 µL of 5 µM iTru Y-yoke adapters. Dispense adapters directly onto bead pellets with multichannel. Do not attempt to mix with pipette.

If using the DynaMag 96-well magnet, it is easiest to add adapters in columns of 8 samples

9. Add 11.2 µL of Ligation master mix to the adapter-moistened bead. Pipette gently to mix.
    a. 2.5 µL buffer, 1.25 µL enzyme*, & 7.5 µL water.

10. Incubate in a Thermocycler at 20°C for 15 minutes.

11. Add 12.5 µL of PEG/NaCl to the 12.5 µL volume, mix, incubate at room temperature for 5-15 minutes.
    - Magnet capture & EtOH wash twice with 80 µL of 80% EtOH.

~Here is where you could incorporate size selection if desired~

12. To dry beads add 12.5 µL of 1X TE Buffer. Incubate at room temperature for 5 minutes. This is the pre-PCR library prep.

There is no need to capture beads or aspirate supernatants. Adapter ligated sample with beads goes into dual-indexing PCR. Keep pre-PCR Library Prep at 4°C while preparing the Dual-Index PCR Plate.

2.4 Dual-Index PCR Library Amplification

13. To a new tube add 12.5 µL of KAPA Hotstart Ready Mix, 1.25 µL of i5 primer, 1.25 µL of i7 primer.

14. Mix 10 µL of the pre-PCR library prep (with speedbeads). Cover with foil seal and spin briefly.

15. Place in thermocycler for 8-12 cycles using the following conditions:

Step		Temperature	Duration
Initial activation step		98°C	45 sec
	Denaturation	***98°C***	***15 sec***
**45 Cycles**	***Annealing***	***60°C***	***30 sec***
	Extension	***72°C***	***30 sec***
Final Extension		72°C	1 min

---

16. Use Qubit or PicoGreen to quantify the PCR product. Capture beads with a magnet and aspirate libraries in supernatant to quantify. The DNA is fully in the TE Buffer at this point. Consider running a HS tape if there is not a significant increase in DNA concentration.

17. If pooling samples for sequencing, combine libraries in equal nanogram amounts by dividing the calculated ng/µL value by the ng of each library desired in the pool.

    Ex. To pool 200 ng of a sample with a concentration of 45ng/µL, divide 200/45 = 4.444~µL

---

18. After pooling libraries, purify with Speedbeads
    a. Add 32 µL of Speedbeads to a 25 µL volume of pooled libraries. Mix gently by pipetting and incubate at room temperature for 5 minutes.
    b. Magnet capture and wash twice with 80 µL of 80% EtOH
    c. To dry beads add 25uL 1X TE or Elution Buffer

19. Libraries are ready to be diluted and loaded onto an Illumina sequencing platform

Data Availability: Sequencing data are available on the sequence read archive (PRJNA1171111). Metadata are available in Table S3 and upon request to Dr. Innocent M. Ali. Custom scripts are available at https://github.com/bailey-lab/pfsmarrt_cameroon_7-6-24.
